# DTFLOW: Inference and Visualization of Single-cell Pseudotime Trajectory Using Diffusion Propagation

**DOI:** 10.1016/j.gpb.2020.08.003

**Published:** 2021-03-02

**Authors:** Jiangyong Wei, Tianshou Zhou, Xinan Zhang, Tianhai Tian

**Affiliations:** 1 College of Science, Huazhong Agricultural University, Wuhan 430070, China; 2 School of Statistics and Mathematics, Zhongnan University of Economics and Law, Wuhan 430073, China; 3 School of Mathematics and Statistics, Sun Yat-sen University, Guangzhou 510275, China; 4 School of Mathematics and Statistics, Central China Normal University, Wuhan 430079, China; 5 School of Mathematics, Monash University, Melbourne, VIC 3800, Australia

**Keywords:** Single-cell heterogeneity, Pseudotime trajectory, Manifold learning, Bhattacharyya kernel

## Abstract

One of the major challenges in single-cell data analysis is the determination of cellular developmental trajectories using single-cell data. Although substantial studies have been conducted in recent years, more effective methods are still strongly needed to infer the developmental processes accurately. This work devises a new method, named DTFLOW, for determining the pseudo-temporal trajectories with multiple branches. DTFLOW consists of two major steps: a new method called **Bhattacharyya kernel** feature decomposition (BKFD) to reduce the data dimensions, and a novel approach named Reverse Searching on k-nearest neighbor graph (RSKG) to identify the multi-branching processes of cellular differentiation. In BKFD, we first establish a stationary distribution for each cell to represent the transition of cellular developmental states based on the random walk with restart algorithm, and then propose a new distance metric for calculating pseudotime of single cells by introducing the Bhattacharyya kernel matrix. The effectiveness of DTFLOW is rigorously examined by using four single-cell datasets. We compare the efficiency of DTFLOW with the published state-of-the-art methods. Simulation results suggest that DTFLOW has superior accuracy and strong robustness properties for constructing pseudotime trajectories. The Python source code of DTFLOW can be freely accessed at https://github.com/statway/DTFLOW.

## Introduction

Recent advances in single-cell technologies have provided powerful tools to measure gene expression levels or protein activities of thousands of single cells in a single experiment. Compared with the traditional experimental studies using bulk samples that average out the responses from a large number of cells, the analysis of cellular aspects at the single-cell level offers promising advantages to investigate the heterogeneity in cellular processes [1]. Since temporal data cannot be collected straightforward, a major step in single-cell studies is to order individual cells according to their progress along the differentiation pathways. The pseudo-temporal data based on the ordered individual cells will ultimately lead to the reconstruction of regulatory networks and cellular differentiation pathways [2]. The investigation of single-cell data is a part of big bio-data studies that will lead to the understanding of diseases and improvement of human health [3], [4].

Since the first algorithm Monocle for the pseudo-temporal ordering [5], a number of data-driven computational methods have been developed to define the relative position of each cell during the differentiation process. The methods for inferring pseudotime trajectories typically consist of two major steps: a dimensionality reduction step and a trajectory modeling step. A class of methods based on the graph theory use the minimum-spanning tree (MST) or shortest path to construct the major structure of trajectories, and then project all single cells onto the major structure to obtain the pseudotime trajectory. These methods include Wanderlust [6], Wishbone [7], TSCAN [8], Monocle [5], Monocle2 [9], Waterfall [10], SCOUT [11], DensityPath [12], and SoptSC [13]. Another class of algorithms employ probabilistic models to obtain the major structure of trajectories, such as Gpfates [14], DeLorean [15], and PhenoPath [16]. In addition, other techniques have been used to develop effective methods, include methods based on differential equations (such as SCOUP [17], Pseudodynamics [18], and PBA [19]), methods using the principal curves (such as Embeddr [20] and Slingshot [21]), and machine learning techniques such as VASC [22]. Usually algorithms based on the graph theory are more efficient, but the accuracy of inference results is susceptible to the noise in datasets. However, methods using probabilistic models or differential equations need high computational cost. Recently, a number of comparison studies have been conducted to examine the performance of these algorithms [23], and more effective methods can be found in the comprehensive literature reviews [24], [25], [26], [27].

Network diffusion, also known as network propagation, has attracted much attention in recent years for identifying disease genes, genetic modules, and drug targets [28]. It has also been used for manifold learning and pseudotime calculation for single-cell data. The nonlinear dimensionality reduction algorithms based on network propagation include DCA and PHATE. Among them, DCA obtains the low-dimensional representation of the high-dimensional dataset by minimizing the Kullback–Leibler divergence between the observed diffusion states and parameterized-multinomial logistic distributions [29], whereas PHATE generates a Markov transition matrix as the diffusion operator and then embeds the operator with the non-metric multi-dimensional scaling (MDS) approach for the visualization of single-cell datasets [30]. In addition, MAGIC alleviates the noises in single-cell datasets and learns the intrinsic biological structures and gene interactions via data diffusion [31]. Diffusion map, as a random walk approach, has also been used to explore the developmental continuum of cell-fate transitions [32], [33]. The diffusion pseudotime (DPT) algorithm defines the diffusion pseudotime distance between two cells using the accumulated Markov transition matrix and determines the ordering of cells based on the distances between a root cell and all other cells [34]. In fact, DPT can obtain the pseudo-temporal ordering results before the dimension reduction step, and thus can detect the subtle changes of gene expression.

Another important issue in single-cell studies is to identify branches in the pseudotime trajectories in order to explore the different developmental pathways. A number of algorithms have been designed to determine the branches and optimal bifurcation points. Among them, DPT determines the branching trajectories by the correlation *versus* anti-correlation relationship of the *dpt* distances between cells [34]. Wishbone identifies two post-bifurcation cell fates using the second eigenvector of a mutual disagreement matrix [7]. In addition, SLICER uses the geodesic entropy metric for branch assignment [35]; TSCAN finds the differentiation structure based on the MST algorithm applied to the cluster centers [8], whereas Monocle2 conducts the branching assignment according to the branches of the DDRTree [9]. However, the majority of these branching detection approaches can identify only one bifurcation point. More sophisticated algorithms are strongly needed to determine the branching processes with multiple bifurcation events.

This work proposes a new method, named DTFLOW, for inferring the pseudotime trajectories using single-cell data. This method uses a new manifold learning method, named Bhattacharyya kernel feature decomposition (BKFD), for the visualization of underlying dataset structure. The innovation of this algorithm includes the usage of the random walk with restart (RWR) method to transform each data point into a discrete distribution and the Bhattacharyya kernel to calculate the similarities between cells. Compared with DPT, RWR includes a free parameter that can be used to tune for better inference results. More importantly, we propose a novel distance metric based on the Bhattacharyya distance to preserve the distances along the manifold. In addition, DTFLOW uses an innovative approach named Reverse Searching on k-nearest neighbor (kNN) graph (RSKG) to identify the underlying multi-branching processes of cellular differentiation. The effectiveness of our proposed algorithm is rigorously examined by the analysis of four single-cell datasets.

## Method

**Figure 1:**
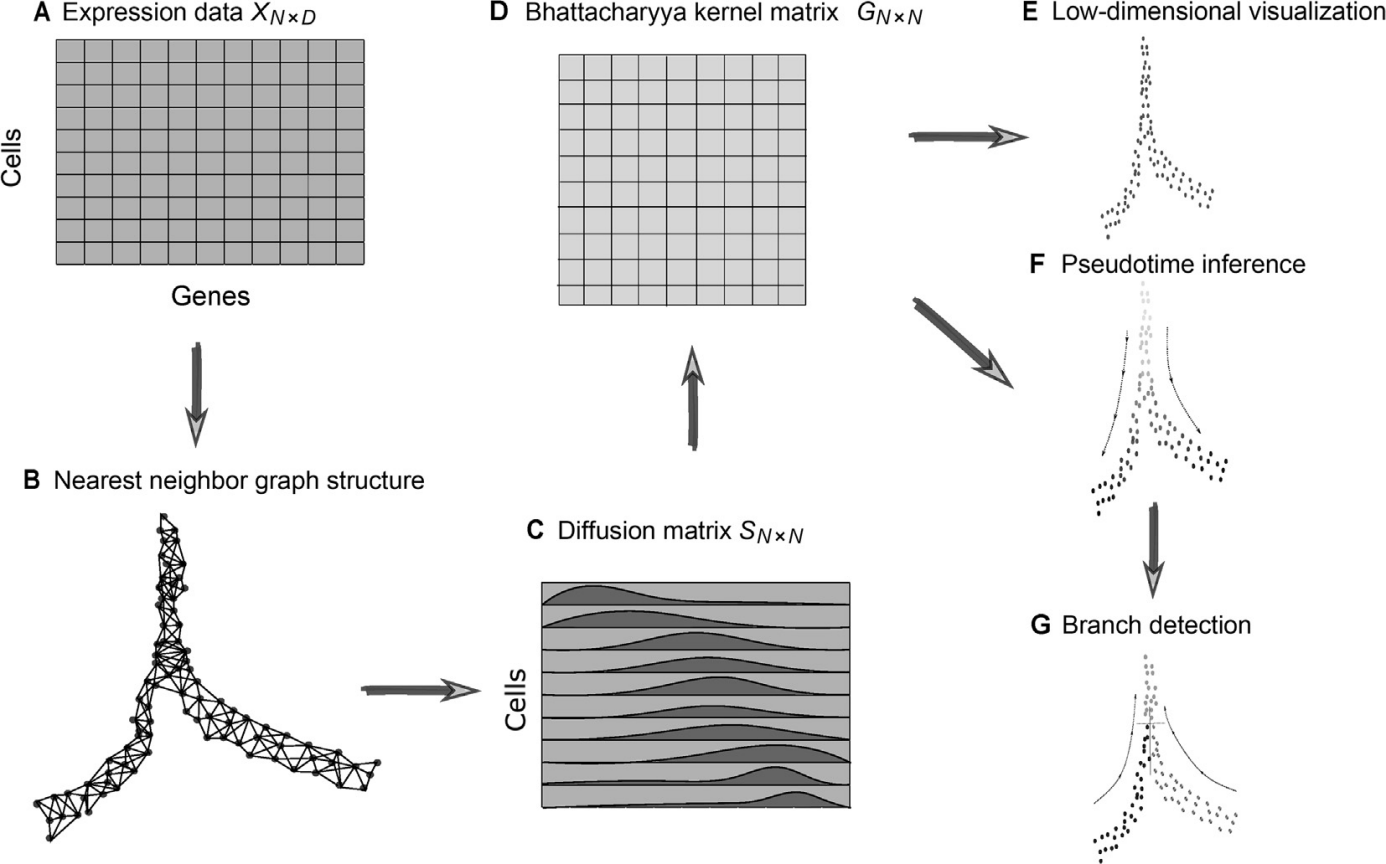
**Overview of DTFLOW algorithm** **A.** Pre-process a single-cell dataset into a gene expression matrix *X*_*N*×*D*_ with *N* cells and *D* genes. **B.** Compute the k nearest neighbors for each cell, get a nearest neighbor graph structure, and then transform the dataset into a Markov transition matrix M. **C.** Use the random walk with restart method to get a diffusion matrix *S*, in which each cell is represented by a discrete distribution vector. **D.** Construct a Bhattacharyya kernel matrix G and a matrix logG based on the properties of the kernel method. **E.** Perform singular value decomposition on logG to get the low-dimensional embedding Y. **F.** Calculate the new distance metric $D_{\mathrm{ri}}$ based on the row of the matrix logG corresponding to the root cell r, and unitize it to get the pseudotime distances T. **G.** Identify the multi-branches of cellular differentiation by reverse searching based on the nearest neighbor graph structure.

This section introduces the proposed DTFLOW for the inference of pseudotime ordering using single-cell data. Figure 1 gives the framework of this algorithm and a brief description of the major steps. The detailed steps can be found in Algorithm 1 in File S1.

### Construction of Markov adjacency matrix

Denote N as the number of cells, D as the number of genes, and $x_{\mathrm{ij}}\in R^{N\times D}$ as the gene expression data. We first find the k most similar neighbors (include itself) of each cell through kNN algorithm based on the pairwise cell–cell Euclidean distance. Using the procedure in [36], we transform the cell–cell Euclidean distances into the symmetric Gaussian kernel weights to represent the affinities/similarities between cells. The transition probability between any two neighbor cells is defined by the Gaussian kernel

(1) $$K\left(x_{i},x_{j}\right)=\exp\left(-\frac{{\left|\left|x_{i}-x_{j}\right|\right|}^{2}}{2\sigma_{i}\sigma_{j}}\right)$$

where $\sigma_{i}$ and $\sigma_{j}$ are the local kernel widths of cell $x_{i}$ and $x_{j}$, respectively. The value of $\sigma_{i}$ is based on the local density with its distance to the k-th nearest neighbor.

If cell $x_{i}$ is a neighbor of $x_{j}$ but $x_{j}$ is not a neighbor of $x_{i}$, we let $K\left(x_{j},x_{i}\right)=K\left(x_{i},x_{j}\right)$ to generate a symmetric kernel matrix. If $x_{i}$ and $x_{j}$ are not the neighbor of each other, $K\left(x_{i},x_{j}\right)=0$. Then we normalize the kernel as

$$\overset{\;}{K}\left(x_{i},x_{j}\right)=\frac{K\left(x_{i},x_{j}\right)}{\sqrt{Z\left(x_{i}\right)Z\left(x_{j}\right)}}$$

(2) $$Z\left(x_{i}\right)=\underset{j}{\sum }K\left(x_{i},x_{j}\right)$$

Finally, we define the Markov transition probability matrix using the normalization over rows, defined by

(3) $$M_{\mathrm{ij}}=\frac{\overset{\sim }{K}\left(x_{i},x_{j}\right)}{\underset{j}{\sum }\overset{\sim }{K}\left(x_{i},x_{j}\right)}$$

### **BKFD**

The RWR algorithm considers each cell as a node, and iteratively calculates the relevance (proximity) score of each node with regard to a given seed node in the kNN graph [37], [38]. At each step, this algorithm selects a move from the current node either to its neighbors with probability p, or return to itself with the restart probability 1-p. Then the distribution vector satisfies the following equation:

(4) $${s_{i}}^{t}=p{s_{i}}^{t-1}M+\left(1-p\right)e_{i},\;0<p<1$$

where ${s_{i}}^{t}$ is an N-dimensional row distribution vector for the visiting probability of each node from the seed node i after t steps, M is defined by Equation (3), and ${s_{i}}^{0}=e_{i}$ is a unit direction row vector, which means that the propagation starts from node i. Thus, the RWR algorithm can be regarded as a more general approach and DPT is a special case of the RWR algorithm (*i.e.*, p=1) (see Section 1 in File S1).

Rather than calculating Equation (4) iteratively in DPT, we introduce the stationary distribution by letting t→∞, which is defined by

(5) $$s_{i}={s_{i}}^{\infty }=\left(1-p\right)e_{i}{\left(I-pM\right)}^{-1}$$

where I is the identity matrix. The diffusion matrix $S={\left[s_{1},\cdots ,s_{N}\right]}^{T}$ is written as

(6) $$S=\left(1-p\right){\left(I-pM\right)}^{-1}$$

The diffusion distribution of each node is a non-vanishing distribution, *i.e.*, $s_{\mathrm{ij}}>0$ and $\sum_{j=1}^{N}s_{\mathrm{ij}}=1$, where element $s_{\mathrm{ij}}$ of matrix S is the similarity score of node j towards node i.

Suppose that p and q are two discrete probability distributions over the same space $\Omega =\{x_{1},\cdots ,x_{N}\}$, and let $p_{i}=p\left(x_{i}\right)$ and $q_{i}=q\left(x_{i}\right)$. Then Bhattacharyya coefficient measures the similarity between p and q, given by

(7) $$BC\left(p,q\right)=\overset{N}{\underset{i=1}{\sum }}\sqrt{p_{i}q_{i}}$$

Based on the definition Equation (7), the Bhattacharyya kernel matrix [39] is defined by

(8) $$G=\sqrt{S}{\sqrt{S}}^{T}={\left[{\langle \sqrt{s_{i}},\sqrt{s_{j}}\rangle }_{i,j=1,\cdots ,N}\right]}_{N\times N}$$

where the square root operation is conducted for every element of the matrix, and $\langle \cdot ,\;\cdot \rangle$ is the inner product of two vectors. Apparently, the diagonal element of matrix G is the inner product of vector $\sqrt{s_{i}}$ and has the value of unit one. Because G is a kernel matrix, its eigenvalues are greater than or equal to 0.

According to Mercer's Theorem, there exists a kernel function k, satisfying that

(9) $$k\left(s_{i},s_{j}\right)=G_{\mathrm{ij}}=\langle \sqrt{s_{i}},\sqrt{s_{j}}\rangle ,\forall i,j\in \left[1,\cdots ,N\right]$$

Based on the properties of kernel functions, we construct a new kernel $k_{1}$ with the mapping operator ϕ, defined by

(10) $$G_{\mathrm{ij}}=k\left(s_{i},s_{j}\right)≜e^{k_{1}\left(s_{i},\;s_{j}\right)}=e^{\langle \phi \left(s_{i}\right),\;\phi \left(s_{j}\right)\rangle }$$

Let $y_{i}=$ $\phi \left(s_{i}\right)$, Equation (10) can be written as $\langle y_{i},y_{j}\rangle =\log G_{\mathrm{ij}}$. Then we rewrite it in the matrix form

(11) $$\log G=Y^{T}Y$$

where $Y={\left[y_{1},\cdots ,y_{N}\right]}^{T}$, and the logarithm operation is applied to every element of matrix G.

Equation (11) is a linear transformation, and we perform the singular value decomposition (SVD) to obtain decomposition

(12) $$\log G=V\Sigma V^{T}$$

where $V\in R^{N\times N}$ is a unitary matrix which satisfies $V^{T}V=I$, and Σ is a diagonal matrix whose elements are the singular values of matrix logG. We use the largest d (positive) singular values to represent the major feature of matrix logG. The d low-dimensional embedding of Y, defined by

(13) $$Y_{d}=V_{d}\Sigma_{d}^{1/2}$$

is used to represent the single-cell dataset. Here $\Sigma_{d}$ is a matrix that includes only the largest d singular values and $V_{d}$ is the corresponding vectors. Normally we use d=2 or d=3 for 2-dimensional or 3-dimensional visualization. Then we use the low-dimensional dataset $Y_{d}$ to visualize the underlying structure of the original high-dimensional single-cell dataset.

### Pseudotime ordering

Note that the Bhattacharyya distance [40] is a measure of similarity between two probability distributions, which is defined by

(14) $$D_{B}\left(i,j\right)=-\log\langle \sqrt{s_{i}},\sqrt{s_{j}}\rangle =-\log G_{\mathrm{ij}}$$

However, this metric does not satisfy the triangle inequality in the inner product space.

To address this issue, we introduce a new distance metric to measure the distance between two cells. From Equation (11), we obtain the distance of two cells i and j as

(15) $${\left|\left|y_{i}-y_{j}\right|\right|}^{2}={\left|\left|y_{i}\right|\right|}^{2}+{\left|\left|y_{j}\right|\right|}^{2}-2\langle y_{i},y_{j}\rangle =-2\log G_{\mathrm{ij}}$$

Since ${\left|\left|y_{i}\right|\right|}^{2}=\log G_{\mathrm{ii}}=0$, we define the new distance metric as

(16) $$D_{\mathrm{ij}}=\left|\left|y_{i}-y_{j}\right|\right|=\sqrt{-2\log G_{\mathrm{ij}}}$$

It can be shown that this new distance satisfies the triangle inequality in the inner product space, which is essentially a kernel distance [41].

If the root cell $x_{r}$ is known, the distance between the root cell and the i-th cell is denoted as $D_{\mathrm{ri}}$, and we use the vector $T_{r}=D_{r,:}$ to denote the pseudotime of single cells. However, if we do not know the root cell in advance, we can select a group of cells as the root cells based on the sum of distances between a particular cell and all other cells. Suppose we select R cells as the group of root cells, the pseudotime of single cells is given by $T_{r}=\sum_{r=1}^{R}D_{r,:}$. Finally, we normalize the pseudotime to values between 0 and 1, given by

(17) $$T=\frac{T_{r}-\min\left\{T_{r}\right\}}{\max\left\{T_{r}\right\}-\min\left\{T_{r}\right\}}$$

### RSKG for branch detection

Based on the constructed kNN graph and pseudotime of each cell, we next propose a new method for branching detection using RSKG. Figure S1 shows a brief description of RSKG for identifying multi-branching processes. The major steps of this algorithm are described in Algorithm 2 and Figure S1.

In this algorithm, n is the minimum number of cells required for forming one sub-branch, T the set of pseudotime of all cells, and A the set of indices array of the kNN graph of all cells. For the id-th cell, $A\left[id\right]$ is the set of its k nearest neighbors. In addition, we use $R_{\mathrm{seq}}$ to store the reverse index ordering based on T. We also use a nested list *prop-groups* to store the candidate sub-branches/groups and a nested list *sub-branches* to store the determined sub-branches. Initially these two nested lists are empty.

This algorithm starts from the cell with the largest pseudotime, whose index $id_{1}$ is the first element in $R_{\mathrm{seq}}$. We put the indexes of its neighbor $A\left[id_{1}\right]$ in the nested list *prop-groups* as the first candidate group. Then we consider the next element $id_{2}$ in $R_{\mathrm{seq}}$ and its neighbor $A\left[id_{2}\right]$. If set $A\left[id_{2}\right]$ has intersections with the list $A\left[id_{1}\right]$, then extend $A\left[id_{2}\right]$ to the list $A\left[id_{1}\right]$ in *prop-groups*. Otherwise, append the list $A\left[id_{2}\right]$ to *prop-groups* as a separate group. The similar procedure is applied to the following elements with index $id_{i}\left(i=3,4,\cdots \right)$.

For the following cells, if $A\left[id_{j}\right]$ has intersections with two or more candidate lists in *prop-groups*, and if the length of two or more intersected lists reaches n, these lists will be moved from *prop-groups* to *sub-branches* and become a determined branch; otherwise, if the length of the merged list does not reach *n*, merge these lists together as one new list in *prop-groups*. If $A\left[id_{j}\right]$ has intersections with lists in both *prop-groups* and *sub-branches*, and if the length of $A\left[id_{j}\right]$ and the intersected list in *prop-groups* reaches n, $A\left[id_{j}\right]$ and the intersected list will be moved from *prop-groups* to *sub-branches* to become a determined branch; otherwise, the elements of $A\left[id_{j}\right]$ and the intersected list in *prop-groups* will be assigned to the branches in *sub-branches* that are closer to them.

### Datasets

Four datasets are used in this work to rigorously examine the performance of the proposed algorithm DTFLOW. The first three datasets are used to test the accuracy and robustness properties of DTFLOW by the inference of pseudo-temporal ordering and dimensionality reduction, while the last one is used to study the efficiency of DTFLOW by the low-dimensional visualization for large datasets. Table 1 provides the summary of these datasets.

**Table 1:** **Summary information of the four datasets used in this study**

The first dataset is the high-throughput RT-PCR dataset [42] that describes the early-stages of the developmental process for mouse embryo (ME). This dataset includes the expression levels of 48 selected genes in 438 single cells at seven different developmental stages, namely from the 1-cell zygote stage to the 64-cell blastocyst stage.

The second dataset is the mouse myeloid progenitor (MMP) MARS-seq dataset that contains 2730 single cells and 3451 informative genes [43]. Note that 10 genes with corrupted names are removed from our analysis based on the pre-processing of Scanpy. In the experimental study, 19 distinct, transcriptionally homogeneous progenitor types/clusters have been identified through an EM-based clustering approach. Among these clusters, clusters 1–6 represent erythroid lineage progenitor (Ery) subpopulations, clusters 7–10 represent common myeloid progenitor (CMP) subpopulations, cluster 11 is for the dendritic cell (DC) fate, clusters 12–18 correspond to granulocyte/macrophage progenitor (GMP) subpopulations, and cluster 19 is the lymphoid lineage progenitors (outlier class) with only 31 cells.

The third dataset is the mouse female gonad (MFG) scRNA-seq dataset that contains 563 single cells and 822 genes at six developmental stages of gonadal differentiation, namely, E10.5, E11.5, E12.5, E13.5, E16.5, and post-natal day 6 (P6) [44].

The final one is the mouse hematopoietic (MH) microwell-seq dataset that contains 51,252 cells and 25,912 genes [45]. After the data pre-processing, the dataset is reduced to 40,210 cells with 100 approximate principal components [46].

## Results

In this section, four datasets are used to evaluate the robustness, accuracy, and efficiency of our proposed algorithm DTFLOW for the inference of pseudotime ordering using single-cell datasets. This work does not include any work for the pre-processing of experimental data. We use the datasets with the same input (namely, the same genes and same single cells) from the published papers directly.

### Accuracy and robustness of DTFLOW

**Figure 2:**
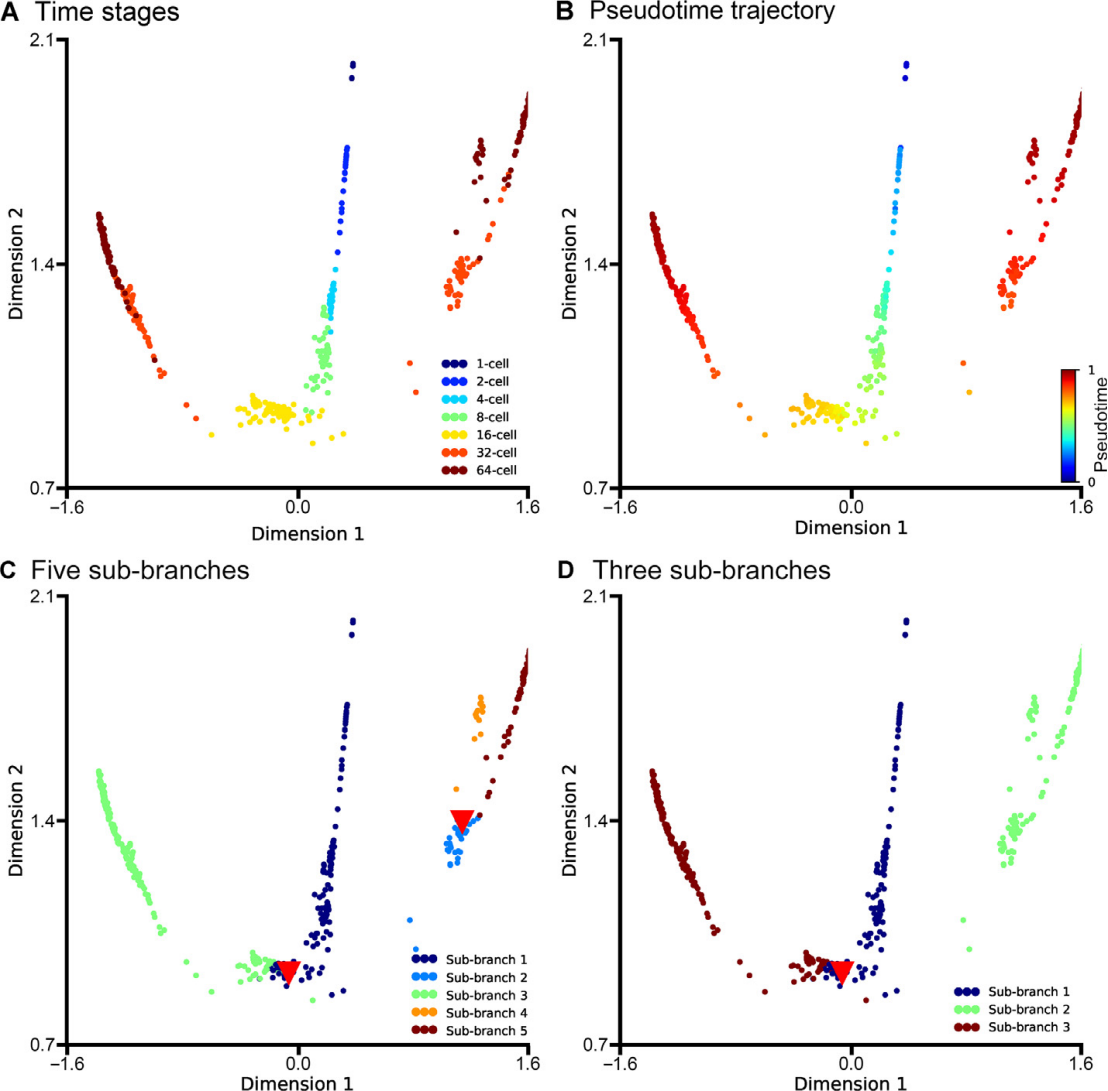
**Developmental trajectories inferred by DTFLOW for the ME dataset** **A.** Visualization of the seven developmental stages in the ME single-cell dataset with 48 genes and 438 single cells [42]. **B.** Visualization of the calculated pseudotime of each single cell, whose values range from 0 to 1. **C.** Visualization of inferred 5 sub-branches when the minimal cell number required for forming a sub-branch satisfies n≤11. **D.** Visualization of inferred 3 sub-branches when the minimal cell number required for forming a sub-branch is larger (n=12-122). Red triangles in (C) and (D) indicate the bifurcation points. ME, mouse embryo.

We first apply DTFLOW to the ME dataset [42] for projecting the 48-dimensional gene expression data into the two-dimensional feature space by using the BKFD algorithm. Figure 2A provides the visualization of single cells at different stages. It clearly reveals the seven developmental stages/labels (namely the 1-cell stage, 2-cell stage, …, and 64-cell stage), which also validates the effectiveness of our proposed dimensional reduction technique. Since not knowing the root cell in the dataset, we select a cell in the initial time stage, which has the largest sum of distances to all other cells, as the root cell. The differentiation process of single cells is characterized by the calculated pseudotime in Figure 2B. These results suggest that the pseudotime of individual cells is recovered successfully.

We also test the influence of the minimal cell number n required for forming a sub-branch. When we set a small value (*i.e.*, n≤11), the individual cells in the lineage process is divided into five sub-branches (Figure 2C). There are two bifurcation points that separate cells into two distinct sub-branches along the differentiation process. Figure 2C shows that the main lineage trajectory contains two major branches and one of them further differentiates into two smaller branches. It also suggests that cell differentiation does not occur in the early stages, but cells in the 32-cell stage differentiate distinctly into trophectoderm (TE) and inner cell mass (ICM). Subsequently, cells in the ICM stage further differentiate into epiblast (EPI) and primitive endoderm (PE) in the 64-cell stage. After the second bifurcating event, the embryo cells are divided into three distinct types: namely TE, PE, and EPI. However, if we use a relatively large value (*i.e.*, n=12-112), the single cells will form only three sub-branches with the first bifurcation event occurred (Figure 2D). The second bifurcation event is not identified since the lengths of sub-branches are less than the minimal cell number n. Note that the distances between cells in our algorithm are calculated based on the high-dimensional Bhattacharyya kernel matrix. However, the data visualized in Figure 2 are the low-dimensional data after the application of SVD.

**Figure 3:**
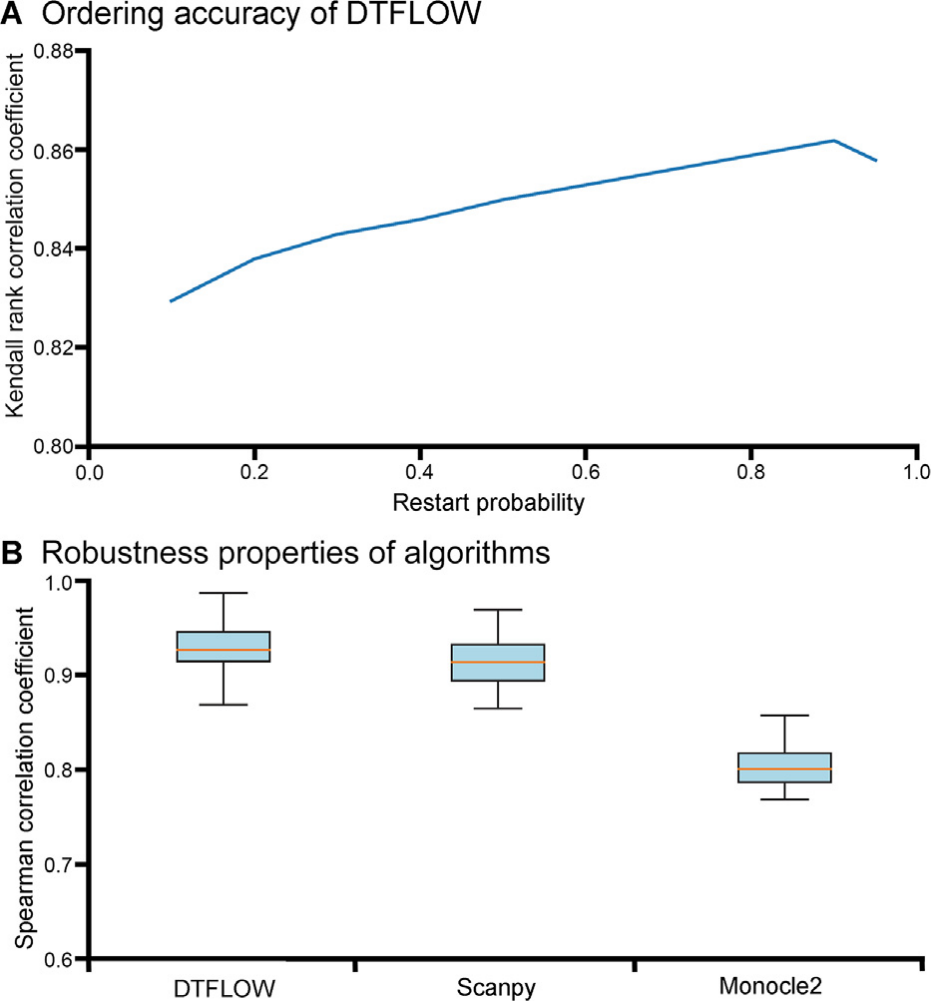
**Accuracy and robustness of three inference methods for the ME dataset** **A.** The accuracy of DTFLOW determined by different values of restart probability p for the ME dataset with 48 genes and 438 single cells [42]. The Kendall rank correlation coefficient is calculated using the stage number of each cell in experimental data and that in the inferred trajectories. **B.** Mean and standard deviation of the Spearman rank correlation coefficient for three inference methods, including DTFLOW, Scanpy, and Monocle2. The correlation coefficient is calculated using the trajectory of randomly sampled 90% of single cells from the whole dataset and that of the whole dataset. Fifty repeated tests are conducted.

In our proposed algorithm, there are two free parameters that should be determined based on the datasets. The first one is the number of closest neighbors k of each data point, which is taken into account for the determination of affinity with classes. To place greater emphasis on the local properties of the manifold structure, a smaller value of k is preferred. Meanwhile, the value of k should also be large enough for the connectivity of the kNN graph. The value of k in BKFD is usually smaller than that in diffusion maps for dimension reduction, which implies that BKFD can capture the local structure of manifold better than diffusion maps. We test different values of k and find that the results are better if k=10, which will be used in this work for analyzing other datasets. The second parameter is the restart probability 1-p that controls the relative influence of both local and global topological structure. To smooth the noise of data, a larger value of p (*i.e.*, a smaller value of 1-p) may be preferred. To test the influence of p, we calculate the pseudo-ordering of single cells using different values of p. We use the Kendall rank correlation coefficient of the inference results to compare the accuracy of the algorithms. Since knowing the stage number of each cell in the experimental data, we determine the stage number of each cell in the inferred trajectories and then calculate the Kendall rank correlation coefficient of these two types of stage numbers. An algorithm has better accuracy if the value of this correlation coefficient is larger. As shown in Figure 3A, the ordering accuracy is better when the value of p is around 0.9. Thus, we use p=0.9 in this work, including the results shown in Figure 2.

To demonstrate the effectiveness of our proposed algorithm, we compare the performance of DTFLOW with two published state-of-the-art methods, namely DPT and Monocle2 [9] (Figures S2 and S3). We use the Python toolkit Scanpy [47] for the implementation of DPT. As shwon in Figure S2C, Scanpy detects only three groups/sub-branches. It fails to identify the number of terminal states correctly, and also obtains the wrong location of bifurcation point. Although Monocle2 identifies three types of the terminal cells correctly (Figure S3C), it does not reveal the intermediate state between state 1 and states 3 and 4 (*i.e.*, the ICM stage) using the dimensional reduction method DDRTree.

For this dataset, we use Kendall rank correlation coefficient to compare the accuracy of these three algorithms. The calculated Kendall rank correlation coefficients are 0.862, 0.796, 0.761 for DTFLOW, Scanpy, and Monocle2, respectively, which suggests that our proposed method has better accuracy than the two published methods.

Figure S4 shows the expression levels of two genes, *Gata3* and *Sox2*, based on the inferred pseudotime using the three methods, which are consistent with the results of visualization. It shows that only DTFLOW detects the ICM stage correctly. The intermediate states of cell development in Monocle2 are not revealed properly possibly because the differences between clusters are amplified by the DDRTree method with the cluster centroids. In addition, DPT uses diffusion maps for dimensional reduction, which may not be sensitive enough to the noise in dataset.

We further conduct the robustness analysis of each algorithm. We first use the whole dataset to infer a trajectory and determine the position of each cell in this trajectory. Then we sample part of the cells from the whole dataset and use the same algorithm to determine the trajectory of cells in the sub-dataset. We calculate the Spearman rank correlation coefficient between the positions of subset cells in the trajectory of the whole dataset and those of the sub-dataset. An algorithm is more robust if the value of the correlation coefficient is larger. We conduct 50 tests to measure the robustness properties of these three algorithms. In each test we randomly sample 90% of cells (*i.e.*, 394 cells) from the dataset and then calculate the Spearman rank correlation coefficient of the pseudotime ordering of the sub-dataset. Then we use the mean and standard deviation of the correlation coefficient based on these 50 test results to measure the robustness properties of algorithms. As shown in Figure 3B, the robustness properties of DTFOLW and Scanpy are better than that of Monocle2. In addition, the variance of correlation coefficients obtained by DTFLOW is smaller than that of Scanpy. These results suggest that the performance of DTFLOW is more stable than the two published methods. To examine the influence of the sampling size, we conduct further robustness test by randomly sampling 80% of cells (*i.e.*, 350 cells) from the dataset. The Spearman rank correlation coefficients shown in Figure S5 are consistent with those shown in Figure 3B.

### Identification of multiple sub-branches

**Figure 4:**
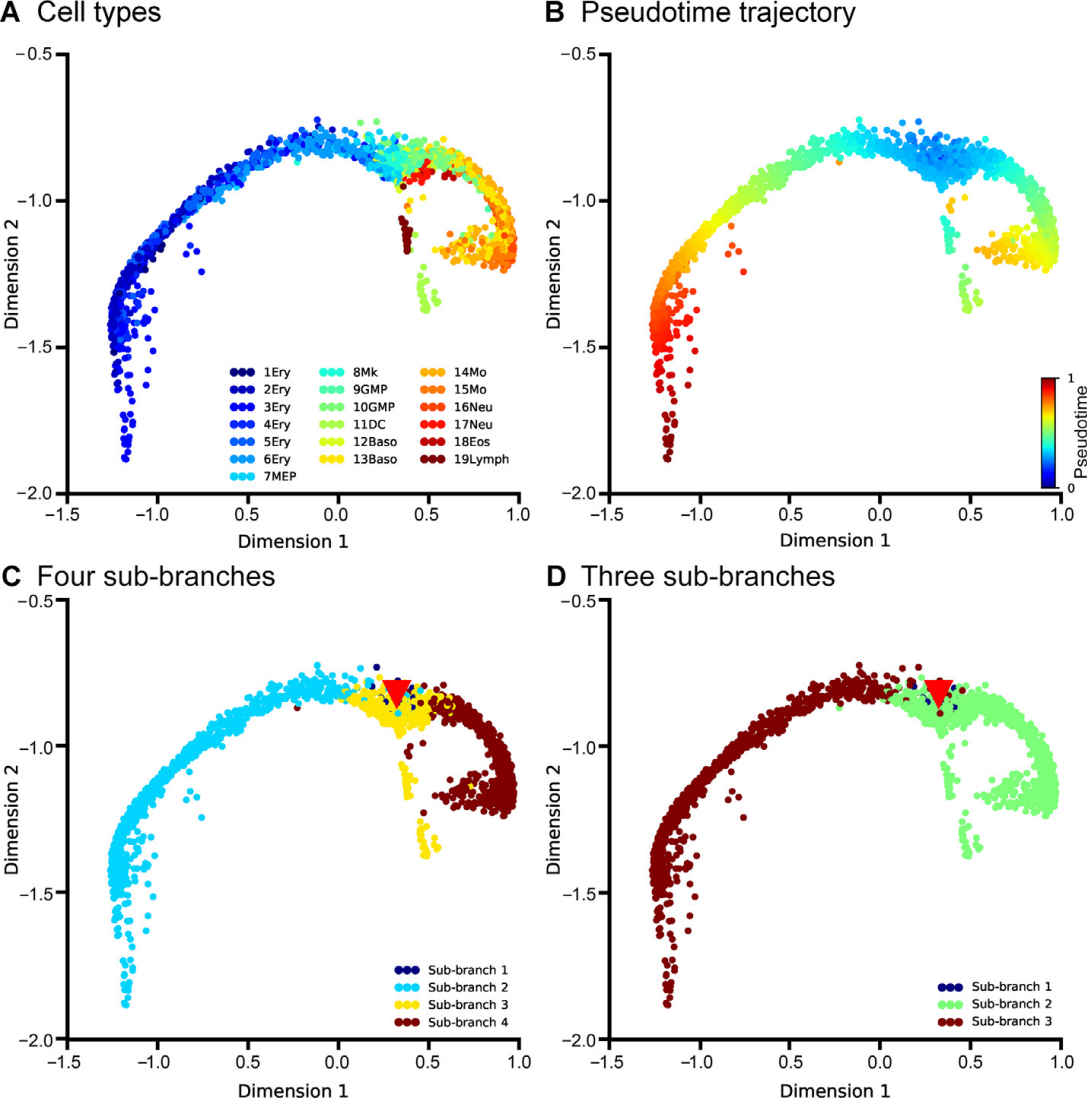
**Developmental trajectories inferred by DTFLOW for the MMP dataset** **A.** Visualization of different cell types in the MMP dataset with 3451 genes and 2730 single cells [43]. **B.** Visualization of inferred temporal trajectory, whose values range from 0 to 1. **C.** Visualization of calculated 4 sub-branches when the minimal cell number required for forming a sub-branch satisfies n=6-22. **D.** Visualization of calculated 3 sub-branches when the minimal cell number required for forming a sub-branch is larger (n=23-124). Red triangles in (C) and (D) indicate the bifurcation points. MMP, mouse myeloid progenitor.

After successfully demonstrating the accuracy and robustness of DTFLOW, we next examine its ability to identify sub-branches. We apply DTFLOW to project the MMP dataset [43] into the two-dimensional feature space. Figure 4A elucidates that CMP and its progenitors (namely, Ery and GMP) are nearly separated in three different regions, while DC and lymphoid cells deviate away from the main differentiation progression process.

To reveal the cellular differentiation process, we select the same cell in [34] as the root cell (*i.e.*, the 840-th cell in cluster 8). Figure 4B demonstrates the pseudotime ordering results from the themyeloid progenitor stage. Figure 4C and D show different branching detection results that are determined by a smaller cell number of n=6-22 and a relatively larger number of n=23-124 for forming sub-branches, respectively. As shonw in Figure 4C, sub-branch 1 contains only a small number of cells. DTFLOW ensures that the pseudotime of each cell in the initial branch is less than that of any other cells in the following sub-branches. Then cells differentiate into three different terminal branches. Sub-branch 2 corresponds to the erythroid evolutionary branch, sub-branch 3 is formed by cells within clusters 11 and 19, and sub-branch 4 corresponds to the GMP branch. This result shows the ability of DTFLOW to identify multiple sub-branches simultaneously. However, when a larger value of n is used, sub-branches 3 and 4 merge together and form a large sub-branch as shown in Figure 4D.

We next compare the branching detection results of DTFLOW, Scanpy, and Monocle2 (Figures S6 and S7). As shown in Figure S6C, Scanpy is also able to identify four sub-branches. However, the root cell identified by Scanpy is not in the initial group, which is unreasonable for the developmental process. Although Monocle2 successfully estimates 12 states (Figure S7C), which is consistent with the experimental observation, it is difficult to analysis the changes of gene expression over time based on this large branch number.

**Figure 5:**
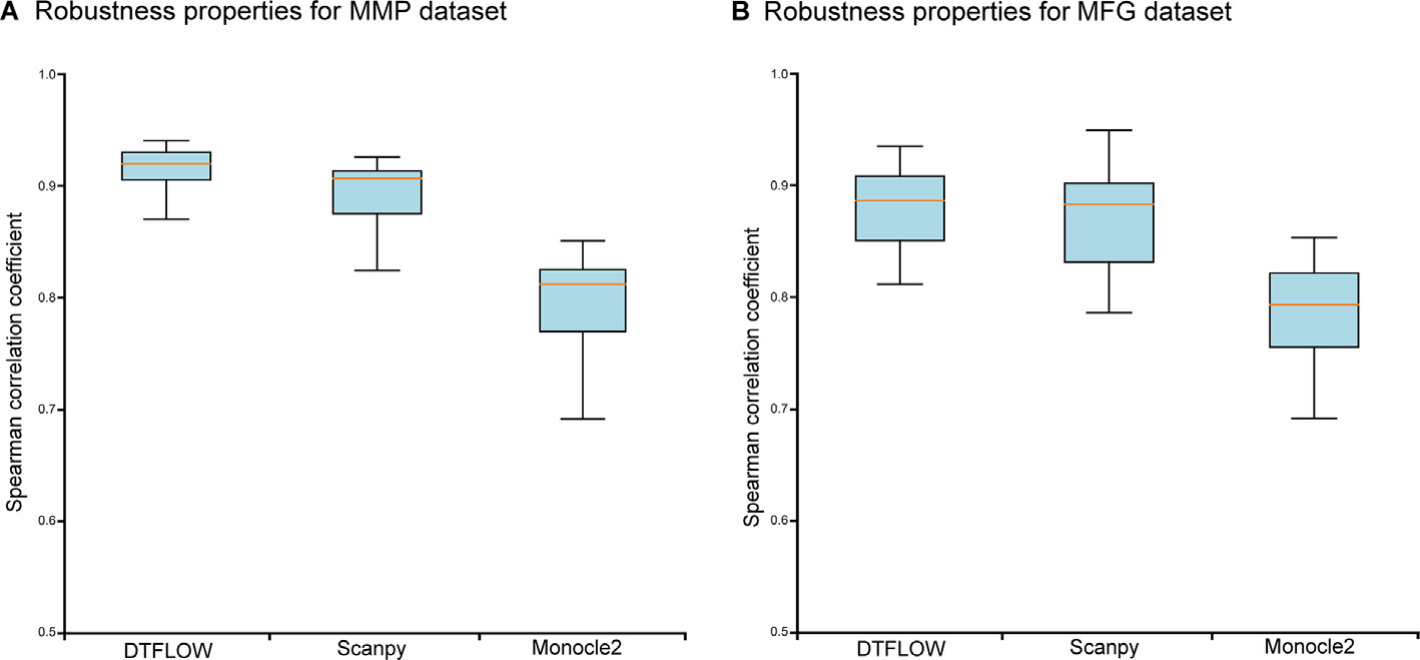
**Robustness properties of three inference methods** **A.** Mean and standard deviation of the Spearman rank correlation coefficient for the MMP dataset with 3451 genes and 2730 single cells [43]. **B.** Mean and standard deviation of the Spearman rank correlation coefficient for the MFG dataset with 822 genes and 563 single cells [44]. The correlation coefficient is calculated using the trajectory of randomly sampled 90% of single cells from the whole dataset and that of the whole dataset. Fifty repeated tests are conducted. MFG, mouse female gonad.

We then carry out robustness analysis of the three methods. For each method, we randomly sample 2500 single cells from 2730 cells and then use the same methods to infer the pseudotime of the selected cells. Then we compare the pseudotime of cells in the sampled set with that of the corresponding cells in the whole dataset by using the Spearman rank correlation coefficient. We conduct 50 repeated tests to measure the robustness property of each method. Figure 5A shows that the robustness properties of DTFOLW and Scanpy are better than that of Monocle2. In addition, the variance of correlation coefficients obtained by DTFLOW is smaller than that of Scanpy. These results suggest that the performance of DTFLOW is more stable than the two published methods.

Note that the gene expression levels in this dataset are not continuous and the three terminal branches have different lengths. To illustrate this, Figure S8 presents the expression visualization of two marker genes, *Elane* and *Klf1*. These marker genes show similar significance in different branches for different dimensionality reduction algorithms. Figure S8A shows that the expression levels of *Elane* are essential for the GMP process while the expression levels of *Klf1* increase gradually on the erythroid branch. The expression trends of these marker genes are different along the constructed trajectories, which provides important information for developing gene regulatory networks.

### Confirmation of accuracy and robustness for DTFLOW

**Figure 6:**
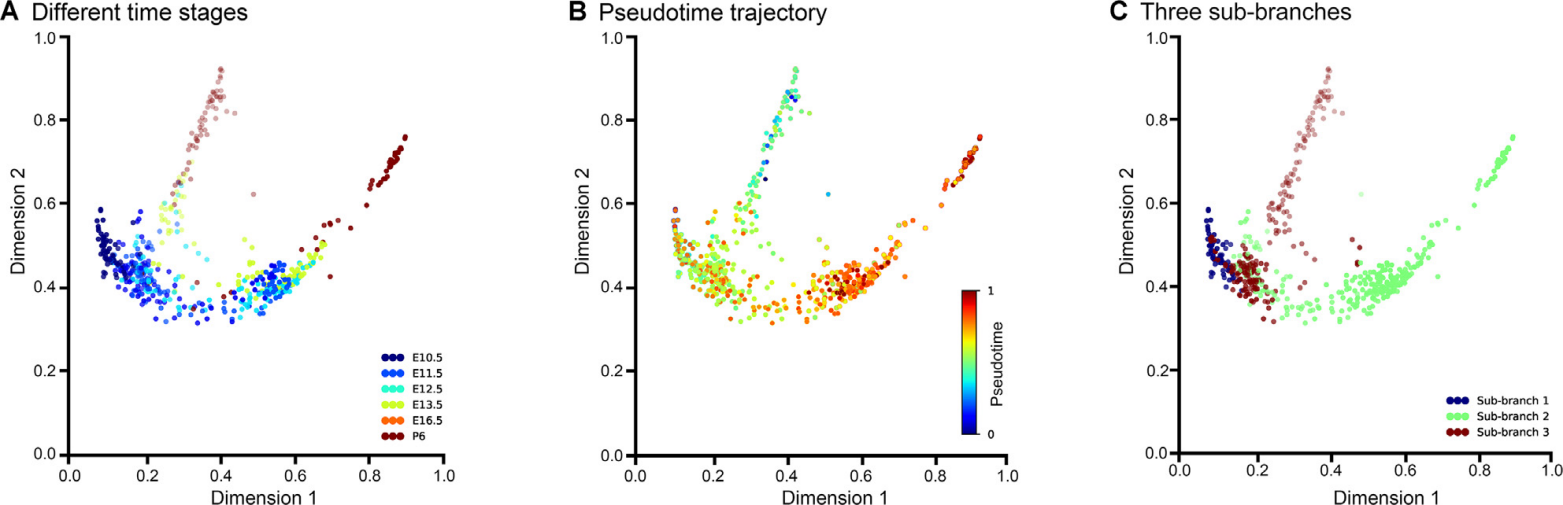
**Developmental trajectories inferred by DTFLOW for the MFG dataset** **A.** Visualization of different cell types in the MFG dataset with 822 genes and 563 single cells [44]. **B.** Visualization of inferred temporal trajectory, whose values range from 0 to 1. **C.** Visualization of the calculated three sub-branches.

To further confirm the accuracy and robustness of DTFLOW, we use a recently published dataset to test the performance of DTFLOW. For the MFG dataset [44], we project it into the 3-dimensional space using our proposed algorithm BKFD. In Figure 6A all the cells are presented by different colors for different stages. It shows that the early progenitor cells subsequently lead to the differentiation to the granulosa cell lineage and stromal progenitor cell lineage in around stages E11.5–E12.5. Figure 6B gives the ordered pseudotime of different single cells and Figure 6C presents the inferred three sub-branches by our proposed DTFLOW.

We first compare the pseudotime ordering accuracy of DTFLOW with Scanpy and Monocle2. Figures S9 and S10 show the analysis results of Scanpy and Monocle2 for this dataset, respectively. The calculated Kendall rank correlation coefficients are 0.761, 0.702, and 0.569 for DTFLOW, Scanpy, and Monocle2, correspondingly. We also compare the robustness properties of DTFOLW with those of Scanpy and Monocle2. We conduct 50 tests to measure the robustness properties of these three algorithms. In each test we randomly sample 90% of cells (*i.e.*, ~ 507 cells) from the dataset and find the pseudotime of these cells. Then we compare the pseudotime of these cells in the sampled set with that of the corresponding cells in the whole dataset by using the Spearman rank correlation. Figure 5B gives the robustness properties of these three methods. Numerical results suggest that the robustness properties of DTFOLW are better than those of Scanpy and Monocle2.

### Effectiveness and efficiency of DTFLOW

**Figure 7:**
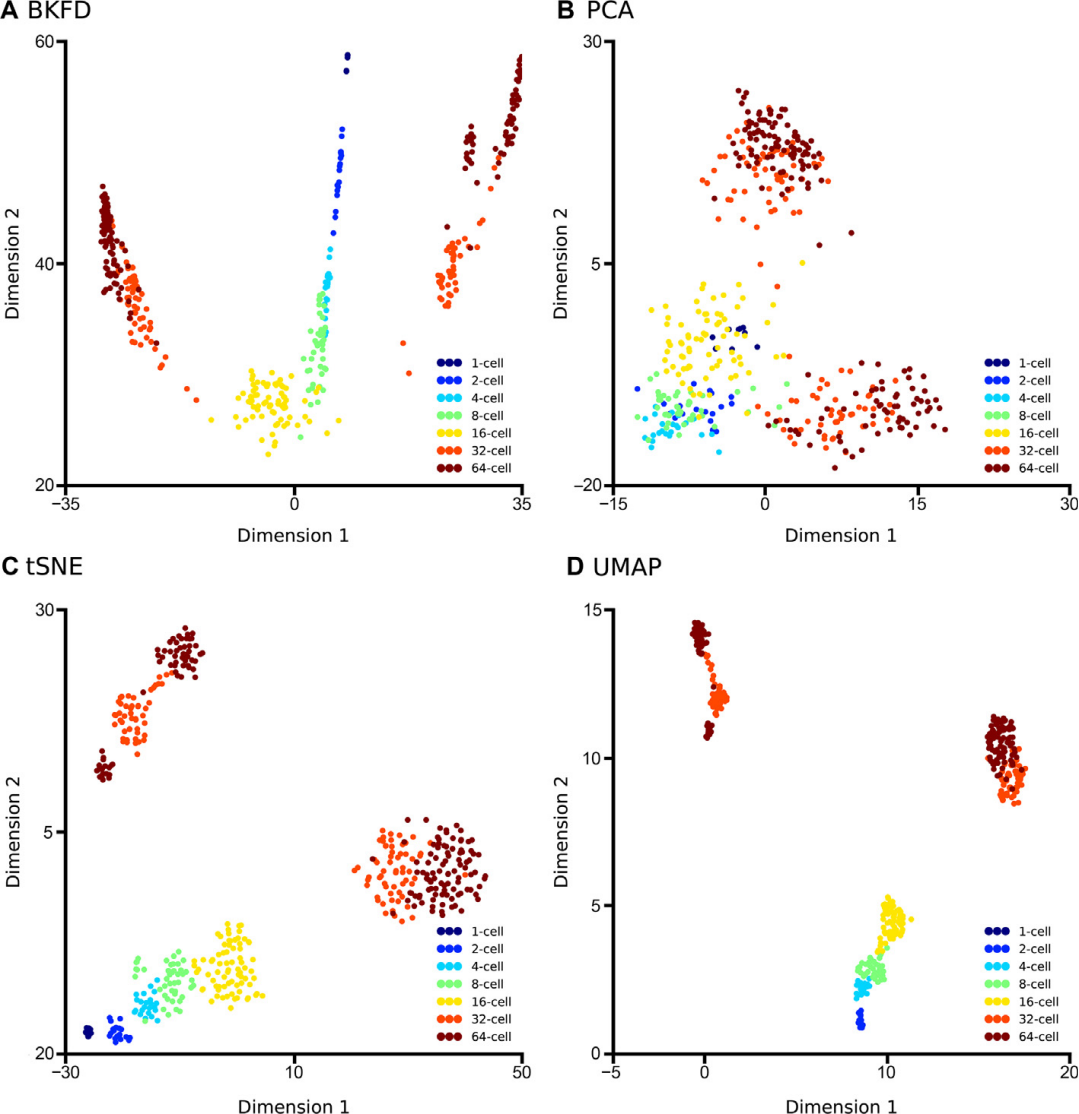
**Dimensional reduction results of four different methods applied to the MEdataset** **A.** BFKD in DTFLOW reasonably represents the cellular developmental process of the ME dataset with 48 genes and 438 single cells [42]. **B.** PCA cannot distinguish differentiation stages very well. **C.** and **D.** tSNE (C) and UMAP (D) do not indicate the cellular developmental process properly. BKFD, Bhattacharyya kernel feature decomposition; PCA, principal component analysis; tSNE, t-distributed stochastic neighbor embedding; UMAP, uniform manifold approximation and projection.

After successfully demonstrating the accuracy and robustness of DTFLOW, the next question is the effectiveness of DTFLOW in dimensional reduction and efficiency for analyzing large-scale single-cell datasets. To answer this question, we first compare our dimensional reduction algorithm BKFD in DTFLOW with several popular and widely used methods, including principal component analysis (PCA), t-distributed stochastic neighbor embedding (tSNE) [48], and uniform manifold approximation and projection (UMAP) [46]. Figure 7 shows the visualization results of the ME single-cell dataset [42]. Based on the idea of diffusion propagation, BKFD represents the cellular development reasonably (Figure 7A). PCA cannot distinguish differentiation stages very well  (Figure 7B). Although tSNE and UMAP can separate different cell types clearly for this dataset, the distance intervals of different cell types are relatively large, which cannot be used to indicate the cellular developmental process properly  (Figure 7C and D).

**Figure 8:**
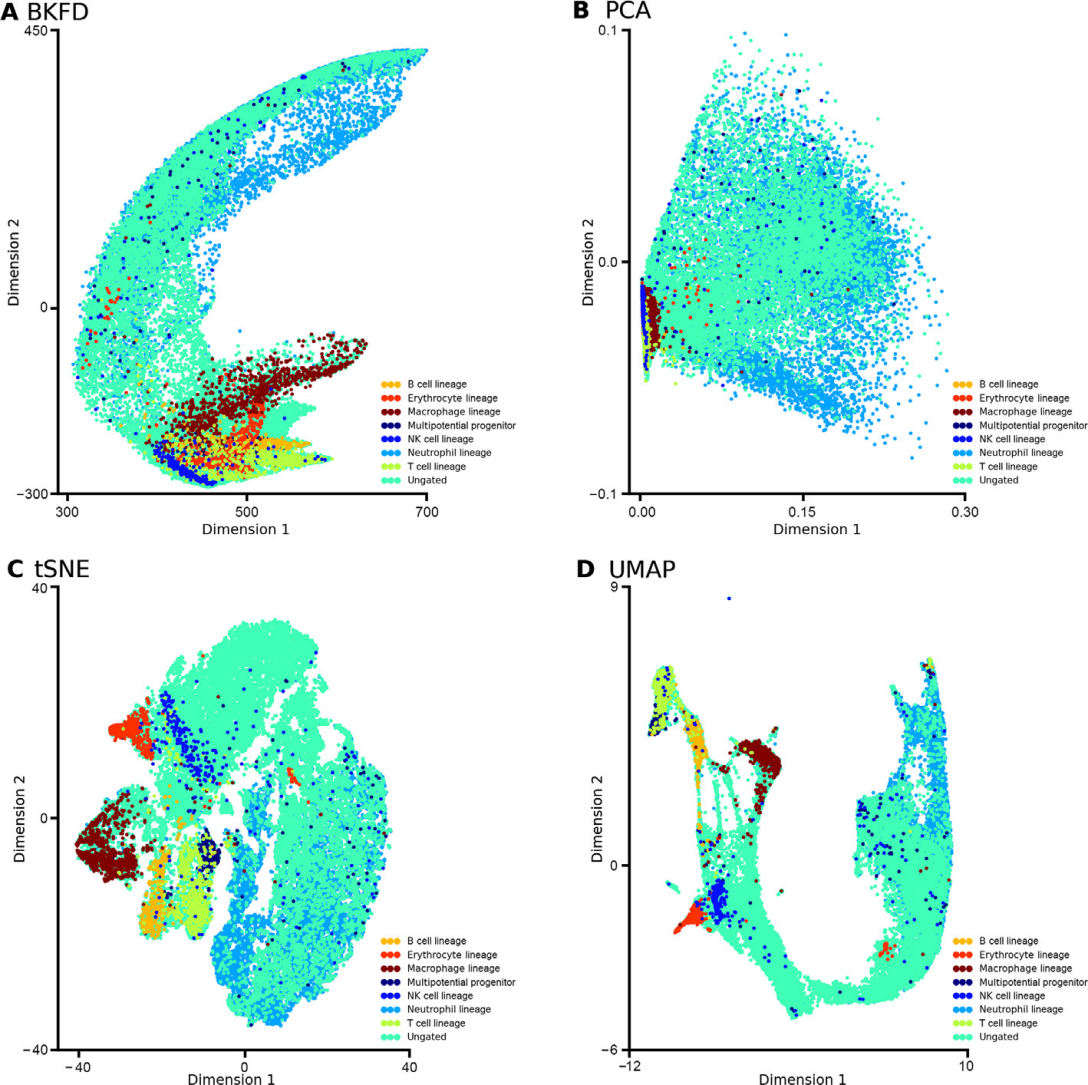
**Dimensional reduction results of four different methods applied to the MH dataset** **A.** BFKD in DTFLOW captures the developmental trajectory in the MH dataset with 40,210 single cells and 25,912 genes [45] in a better way. **B.** PCA cannot show good visualization results with distinguishable cell clusters. **C.** and **D.** tSNE (C) and UMAP (D) can distinguish different cell types clearly. MH, mouse hematopoietic.

To test the efficiency, we next apply BKFD in DTFLOW to a large-scale dataset, which is the MH dataset that contains 40,210 cells and 25,912 genes [45]. We use four methods for dimensional reduction. Figure 8 shows the visualization results of this dataset with eight major cell clusters. It suggests that BKFD can capture the developmental trajectories in a better way (Figure 8A). In addition, tSNE and UMAP can also distinguish different cell types clearly (Figure 8C and D). However, PCA cannot show good visualization results with distinguishable cell clusters for this dataset (Figure 8B). Numerical results show that our designed dimensional reduction algorithm in DTFLOW has similar or better performance than the three widely used algorithms.

## Discussion

In this study, we propose a new method DTFLOW for conducting pseudotime analysis of single-cell data. This method has two major steps: a new dimension reduction method BKFD and a novel approach RSKG to identify the underlying multi-branching processes of cellular differentiation. In BKFD we first establish a stationary distribution for each cell to represent the transition of cellular developmental states based on the RWR algorithm, and then propose a new Bhattacharyya kernel matrix to measure the distances between the distributions obtained by RWR. We use this novel distance metric to calculate the pseudotime distances between single cells before dimension reduction. Thus, our method can reduce the information loss in data processing and increase the inference accuracy. The combination of RWR and the Bhattacharyya kernel matrix shows great power to explore the global structure of the developmental processes using single-cell datasets. In addition, we design the RSKG algorithm to identify the multi-branching of cellular processes. Four datasets are used to compare the accuracy, robustness, and branch detection of the proposed algorithm with two popular published methods. Inference results suggest that our proposed method is more accurate and robust than the published algorithms for inferring the pseudotime trajectories of single cells.

The RWR algorithm is a popular method to estimate the global similarity between a particular node with other nodes in the graph structure. We use this method to transform the data of each node to a stationary discrete distribution. Thus, the input space becomes a set of distributions over the same space. The performance of DTFLOW is affected by the choice of Gaussian kernel function, the number of closest neighbors k, and the restart probability 1-p in the RWR algorithm. Although we have examined the performance of the proposed algorithm by using four datasets, the values of these parameters may vary from dataset to dataset. In addition, BKFD uses the same restart probability for all the nodes, and this may limit the effectiveness of random walk [49]. It is still a challenge to express each cell by a distribution vector in a better way, which needs to be studied in the future.

The continuously topological structure of cellular developmental processes can be analyzed by using the kNN graph, which lays the basis of the DTFLOW algorithm. The kNN graph describes the similarities between a cell and its neighbor cells, and has been used twice in the proposed method, namely the definition of transition probability matrix, which leads to the low-dimensional visualization via the Bhattacharyya kernel matrix, and the determination of branching processes in the RSKG algorithm. The new branch detection algorithm identifies the sub-branches through reverse searching on the sequence of indices ordering and provides biological insights into developmental bifurcations. It can ensure that the sub-branches can be connected through the kNN graph, which in turn also verifies its consistency with the pseudotime inference and visualization results of BKFD.

Scalability is an important issue for the implementation of algorithms. Our algorithm is connected to the dataset size (*i.e.*, the number of cells) in two major steps: the computation of matrix *S* by finding the inverse of matrix (*I* − *pM*) in Equation (6), and the SVD computation in Equation (12). In this study we consider four datasets with cell numbers of 438, 2730, 563, and 40,210, respectively. The computational time of our algorithm is 0.224 s, 11.65 s, 0.246 s, and 3108.35 s on a Lenovo ThinkPad P53 mobile workstation with 2.6 GHz CPU for these four datasets, respectively, which is close to the computing time of other algorithms. In addition, the computing time is in the order of $O(N^{2})$ in terms of the dataset size N, which suggests our program is scalable to dataset size.

In summary, the proposed algorithm DTFLOW provides a new framework for inferring the pseudotime of single cells. Numerical results suggest that it is a power tool for the inference and visualization of cellular developmental trajectories. Potential future work may include the selection of parameters in the proposed method in order to achieve optimal performance in single-cell data analysis.

## Code availability

The Python source code of DTFLOW can be freely accessed at https://github.com/statway/DTFLOW.

## CRediT author statement

**Jiangyong Wei:** Methodology, Software, Formal analysis, Investigation, Resources, Data curation, Writing - original draft, Writing - review & editing, Funding acquisition. **Tianshou Zhou:** Formal analysis, Resources, Funding acquisition. **Xinan Zhang:** Formal analysis, Resources, Funding acquisition. **Tianhai Tian:** Conceptualization, Software, Formal analysis, Investigation, Resources, Writing - review & editing. All authors read and approved the final manuscript.

## Competing interests

The authors have declared no competing interests.

## Supplementary material

Supplementary File S1 **DTFLOW algorithm**.

Supplementary **Figure S1** **Branch detection algorithm by reverse-searching in the kNN graph**. The detailed description of this algorithm is in the main text.

Supplementary Figure S**2** **Visualization of the ME dataset by using Scanpy**. **A****.** Visualization of seven developmental stages in the ME dataset with 48 genes and 438 single cells [43]. **B****.** Visualization of pseudotime of each single cell, whose values range from 0 to 1. **C****.** Visualization of 3 groups/sub-branches.

Supplementary Figure S**3** **Visualization of the ME dataset by using Monocle2. A****.** Visualization of seven developmental stages in the ME dataset with 48 genes and 438 single cells [43]. **B****.** Visualization of pseudotime of each single cell, whose values range from 0 to 1. **C****.** Visualization of 4 states/sub-branches.

Supplementary Figure S**4** **Trajectories for the expression values of two genes of the ME dataset**. **A****.** Trajectories of gene *Gata3* in the ME dataset with 48 genes and 438 single cells [43]. **B****.** Trajectories of gene *Sox2* in the ME dataset. The ordered values from the ME dataset are plotted along pseudotime obtained by using the three inference methods. The lines correspond to the results by using the Gaussion process regression for each branch.

Supplementary Figure S**5** **Robustness properties of three inference methods for the ME dataset**. We randomly sample 80% of single cells from the whole dataset with 48 genes and 438 single cells [43] and compare the ordering results of the subset with those of the whole dataset by using the Kendall rank correlation coefficient. Means and standard deviations of 50 repeated tests are presented.

Supplementary Figure S**6** **Visualization of the MMP dataset using Scanpy**. **A****.** Visualization of 19 cell clusters in the MMP dataset with 3451 genes and 2730 single cells [44]. **B****.** Visualization of pseudotime of each single cell, whose values range from 0 to 1. **C.** Visualization of 4 groups/sub-branches.

Supplementary Figure S**7** **Visualization of the MMP dataset using Monocle2**. **A****.** Visualization of 10 cell types in the MMP dataset with 3451 genes and 2730 single cells [44]. **B****.** Visualization of pseudotime of each single cell, whose values range from 0 to 1. **C.** Visualization of 12 states/sub-branches.

Supplementary Figure S**8** **Trajectories for the expression values of two genes of the MMP dataset**. **A****.** Trajectories of gene *Elane* in the MMP dataset with 3451 genes and 2730 single cells [44]. **B****.** Trajectories of gene *Klf1* in the MMP dataset. The ordered values from the MMP dataset are plotted along pseudotime obtained by using the three inference methods. The lines correspond to the results by using the Gaussian process regression for each branch.

Supplementary Figure S**9** **Visualization of the MFG dataset using Scanpy**. **A****.** Visualization of 6 cell clusters in the MFG dataset with 822 genes and 563 single cells [45]. **B****.** Visualization of pseudotime of each single cell, whose values range from 0 to 1. **C****.** Visualization of 4 groups/sub-branches.

Supplementary Figure S**10** **Visualization of the MFG dataset using Monocle2**. **A****.** Visualization of 6 cell clusters in the MFG dataset with 822 genes and 563 single cells [45]. **B****.** Visualization of pseudotime of each single cell, whose values range from 0 to 1. **C****.** Visualization of 3 states/sub-branches.
