# Supplementary material for: DTFLOW: Inference and Visualization of Single-cell Pseudotime Trajectory Using Diffusion Propagation
Source: Genomics Proteomics Bioinformatics. 2021 Mar 2;19(2):306–18. doi: 10.1016/j.gpb.2020.08.003 (PMC8602766; doi:10.1016/j.gpb.2020.08.003)
Supplement: Supplementary File S1 — DTFLOW algorithm. [file mmc1.docx]

**File S1 DTFLOW algorithm**

**Section 1 Comparison between DPT and DTFLOW**

In this section, we show that DPT is a special case of our proposed algorithm DTFLOW. Note that DPT and DTFLOW use the similar propagation process. DPT provides the (time independent) "path integral" by



 (S1)

where $f\left( t \right)\in R^{n}$ is a probability density. Then the diffusion process of DPT is given by

$S=\sum_{k=1}^{\infty} \left( M-\psi_{0}\psi_{0}^{T} \right)^{k}=\left( I-\left( M-\psi_{0}\psi_{0}^{T} \right) \right)^{-1}-I,$ (S2)

where the eigenvector $\psi_{0}$ is corresponding to the eigenvalue 1 of the transition matrix $M$. The diffusion pseudotime distance metric *dpt* is given by

$dpt^{2}\left( x_{i},x_{j} \right)=\left| \left| S\left( x_{i},\cdot\right)-S\left( x_{j},\cdot\right) \right| \right|^{2},$ (S3)

which implies that it is also a kernel distance based on one kernel matrix $SS^{T}$.

For the DTFLOW algorithm, we have derived the expression of the diffusion matrix S in the main text, namely Equation 6

$S=\left( 1-p \right)\left( I-pM \right)^{-1}.$ (S4)

This equation can also be written as

$$S=\left( 1-p \right)\left( I+pM+p^{2}M^{2}+\cdots+p^{t}M^{t}+\cdots\right)$$

$=\left( 1-p \right)\left( I+\sum_{t=1}^{\infty} p^{t}M^{t} \right) =\left( 1-p \right)\left( I-pM \right)^{-1}.$ (4)

Thus, DTFLOW can use parameter $p$ to control the propagation procedure. This may be the reason that the accuracy of DTFLOW is better than DPT.

**Section 2 The DTFLOW algorithm**

This section provides the detailed description of the proposed algorithm DTFLOW.

This algorithm includes two parts. The first part is the Bhattacharyya kernel feature decomposition (BKFD), which is designed for dimensional reduction. The second part is the branching detection algorithm by reverse searching in the kNN graph (RSKG).

**Algorithm 1 DTFLOW**

**Part 1** BKFD

**Input:** Single-cell data matrix $X_{N\times D}$, number of neighborhoods $k$, restart probability $1-p$, root cell $r$.

Output: Low-dimensional structure $Y_{d}$ and pseudotime distances $T$.

1. Compute the $k\text{-nearest}$ neighbors for each cell $X_{i,:}$ to get a nearest neighbor graph structure.
2. $K\leftarrow$ transform the cell-cell. nearest neighbor distances of graph structure into a symmetric Gaussian kernel weight matrix.
3. $M\leftarrow$ normalize $K$ to get a Markov transition matrix.
4. Using the random walk with restart method to get one diffusion matrix $S\leftarrow\left( 1-p \right)\left( I-pM \right)^{-1}$.
5. Construct a Bhattacharyya kernel matrix $G\leftarrow\sqrt{S}\sqrt{S}^{T}$.
6. $\log G\leftarrow$ construct a new kernel matrix based on the property of kernel method.
7. $Y_{d}\leftarrow$ perform singular value decomposition on $\log G$.
8. $T\leftarrow$ for each cell $i$ and the root cell $r$, calcualate new distance metric $D_{ri}=\sqrt{-2\log G_{ij}}$,then unitize the distance metric.

**Part 2**  Branch detection by RSKG

**Input:** Indices array $A$ of kNN graph, pseudotime of single cells $\mathbf{T}$, the minimum number $n$ of cells for a sub-branch.

**Output:** Sub-branch classification $\mathbf{B}_{\mathbf{vec}}$ of single cells.

$R_{\mathrm{seq}}\leftarrow$ the reverse indices ordering deduced by $\mathbf{T}$.

For $\mathrm{id}$ in $R_{\mathrm{seq}}$:

$A\left[ \mathrm{id} \right]\leftarrow$ $k$-nearest neighbours of the $id$-th cell as one list group

If $A[id]$ has no intersection with any list in prop-groups:

Append $A[id]$ to prop-groups

Else

If $A\left[ \mathrm{id} \right]$ has intersections with only one list in prop-groups:

Extend $A[id]$ to that list of prop-groups

Else

If $A[id]$ has intersection with two or more list of prop-groups:

Pop these intersected lists in prop-groups.

If At most one list has length $\geq n$:

Merge these intersected list together as one new list and append it to prop-groups

Else

Let cell $b_{r}$ with min pseudotime in the intersection as bifurcation point.

Append the lists whose lengths $\geq n$ and only the cells whose pseudotime $>b_{r}$to sub-branches.

Extend cells whose pseudotime $>b_{r}$ in the other list to its nearest sub-branch in sub-branches

Merge $b_{r}$ and the cells whose pseudotime $<b_{r}$ to one list and append the list to prop-groups.

If $A[id]$ has intersection with both prop-groups and sub-branches:

Let cell $b_{r}$ with min pseudotime in the intersection as bifurcation point.

Pop these intersected lists in prop-groups….

Append the lists whose lengths $\geq n$ and only the cells whose pseudotime $>b_{r}$ to sub-branches.

Extend cells whose pseudotime $>b_{r}$ in other list of prop-groups to their nearest sub-branches in the sub-branches.

Merge the cells whose pseudotime $<b_{r}$ to one list and append the list to prop-groups….

Assign the remaining lists in prop-groups to sub-branches, then get $B_{\mathrm{vec}}$ based on sub-branches.
