# Supplementary figures and images for: DTFLOW: Inference and Visualization of Single-cell Pseudotime Trajectory Using Diffusion Propagation
Source: Genomics Proteomics Bioinformatics. 2021 Mar 2;19(2):306–18. doi: 10.1016/j.gpb.2020.08.003 (PMC8602766; doi:10.1016/j.gpb.2020.08.003)

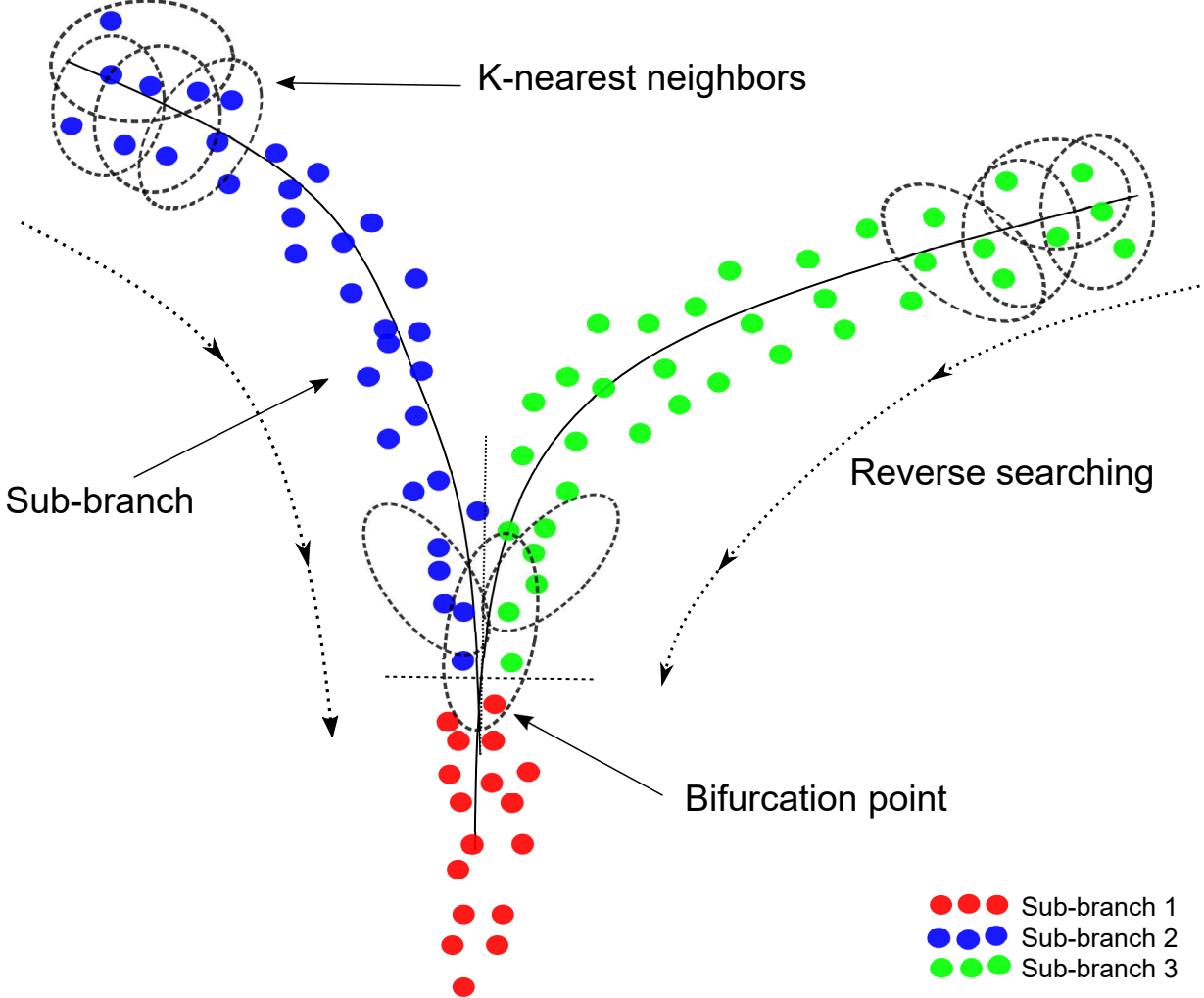

Supplement: Supplementary Figure S1 — Branch detection algorithm by reverse-searching in the kNN graph. The detailed description of this algorithm is in the main text. [file mmc2.pdf]

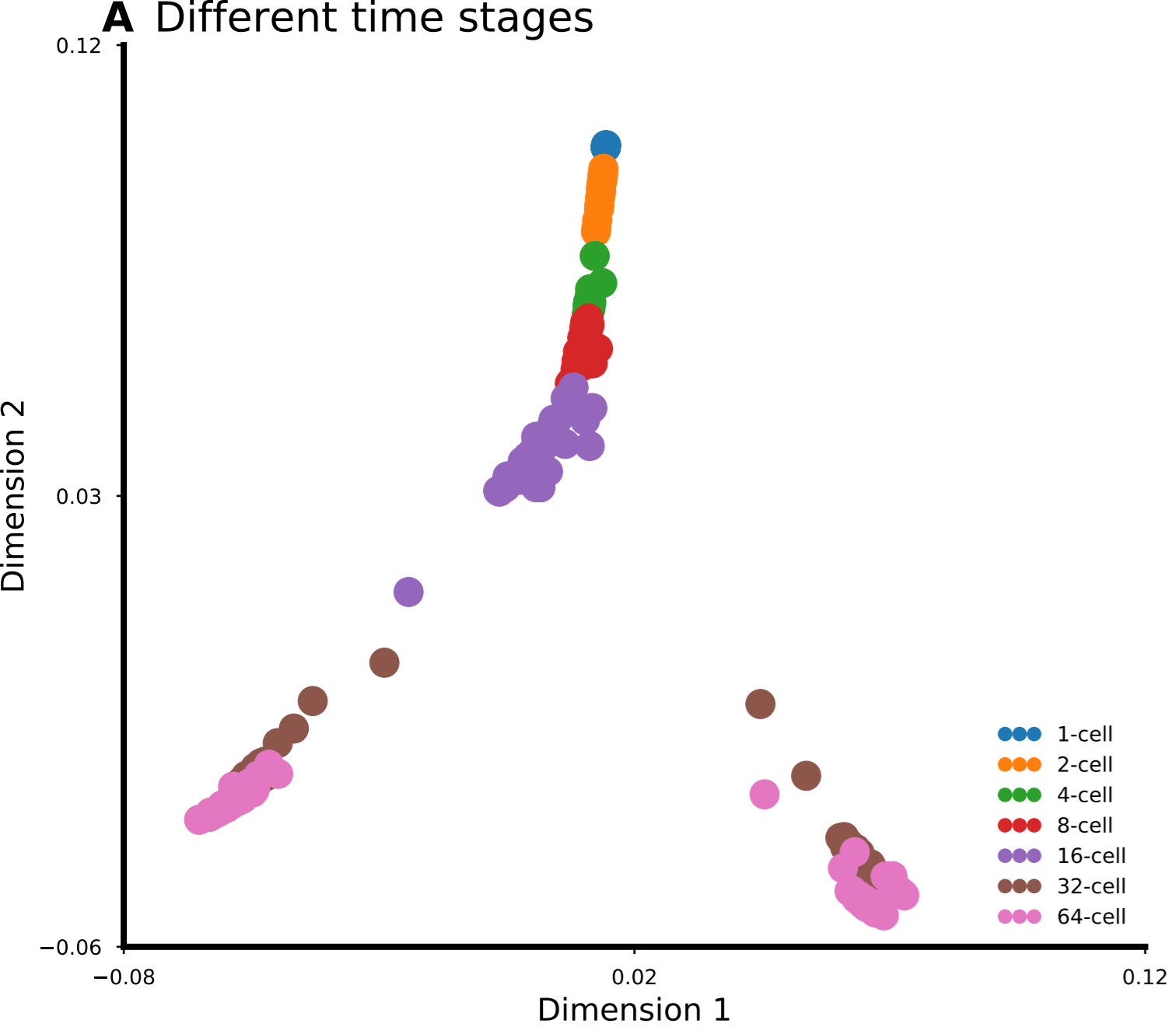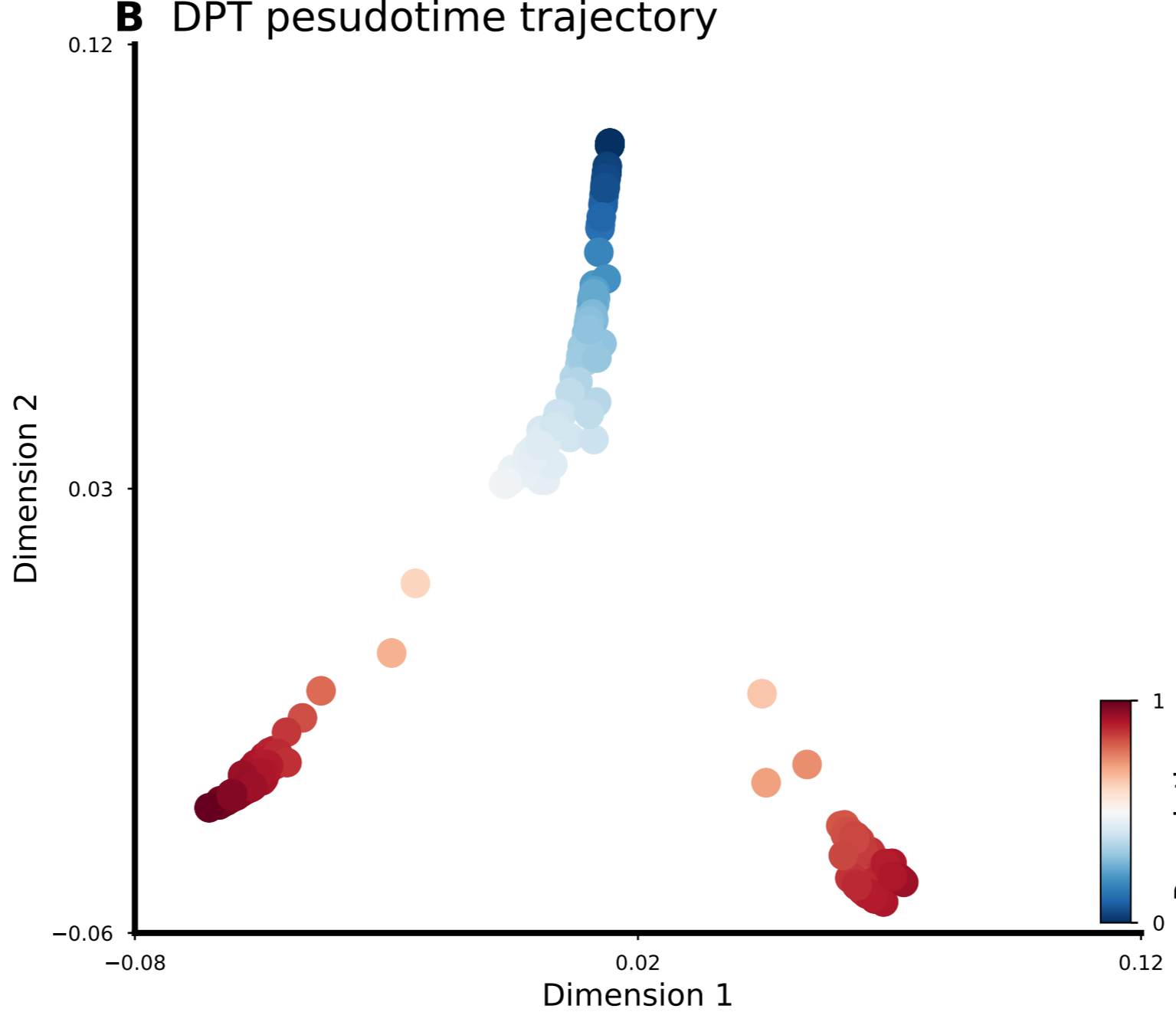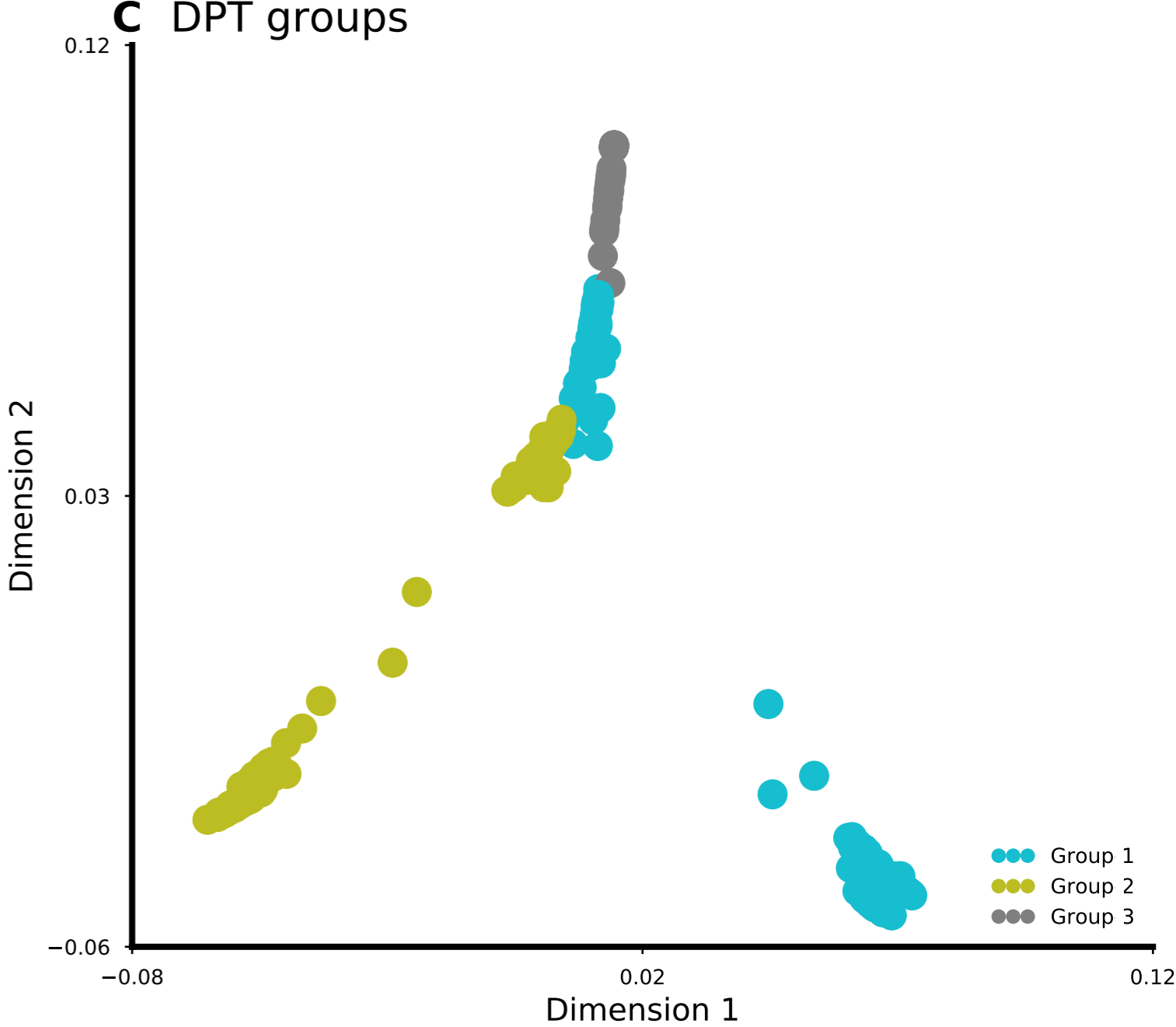

Supplement: Supplementary Figure S2 — Visualization of the ME dataset by using Scanpy. A. Visualization of seven developmental stages in the ME dataset with 48 genes and 438 single cells [43]. B. Visualization of pseudotime of each single cell, whose values range from 0 to 1. C. Visualization of 3 groups/sub-branches. [file mmc3.pdf]

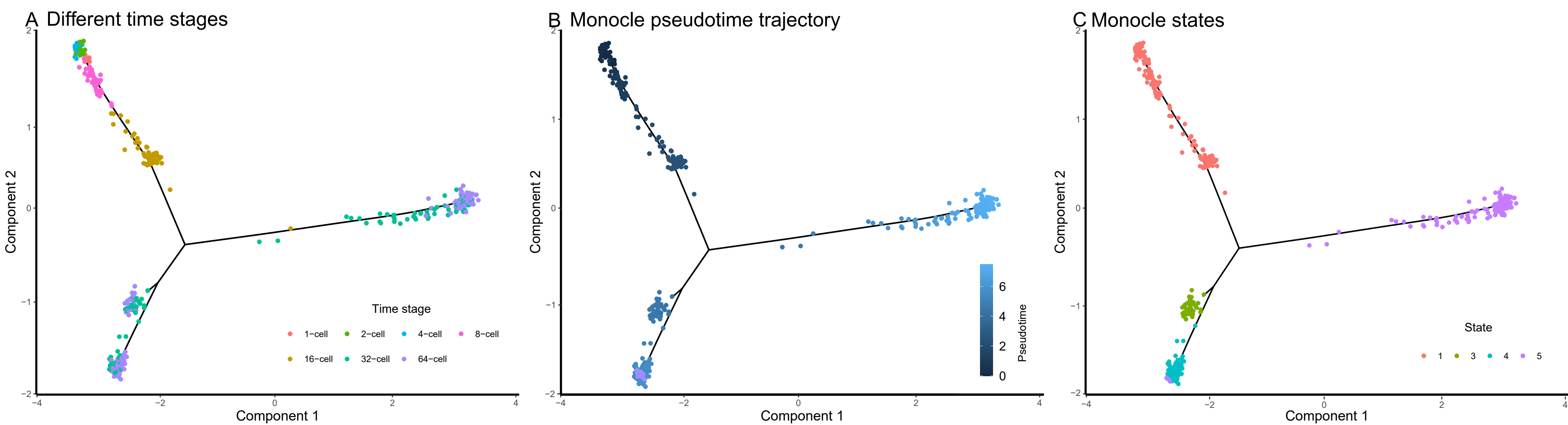

Supplement: Supplementary Figure S3 — Visualization of the ME dataset by using Monocle2. A. Visualization of seven developmental stages in the ME dataset with 48 genes and 438 single cells [43]. B. Visualization of pseudotime of each single cell, whose values range from 0 to 1. C. Visualization of 4 states/sub-branches. [file mmc4.pdf]

## DTFLOW

**A** *Gata3*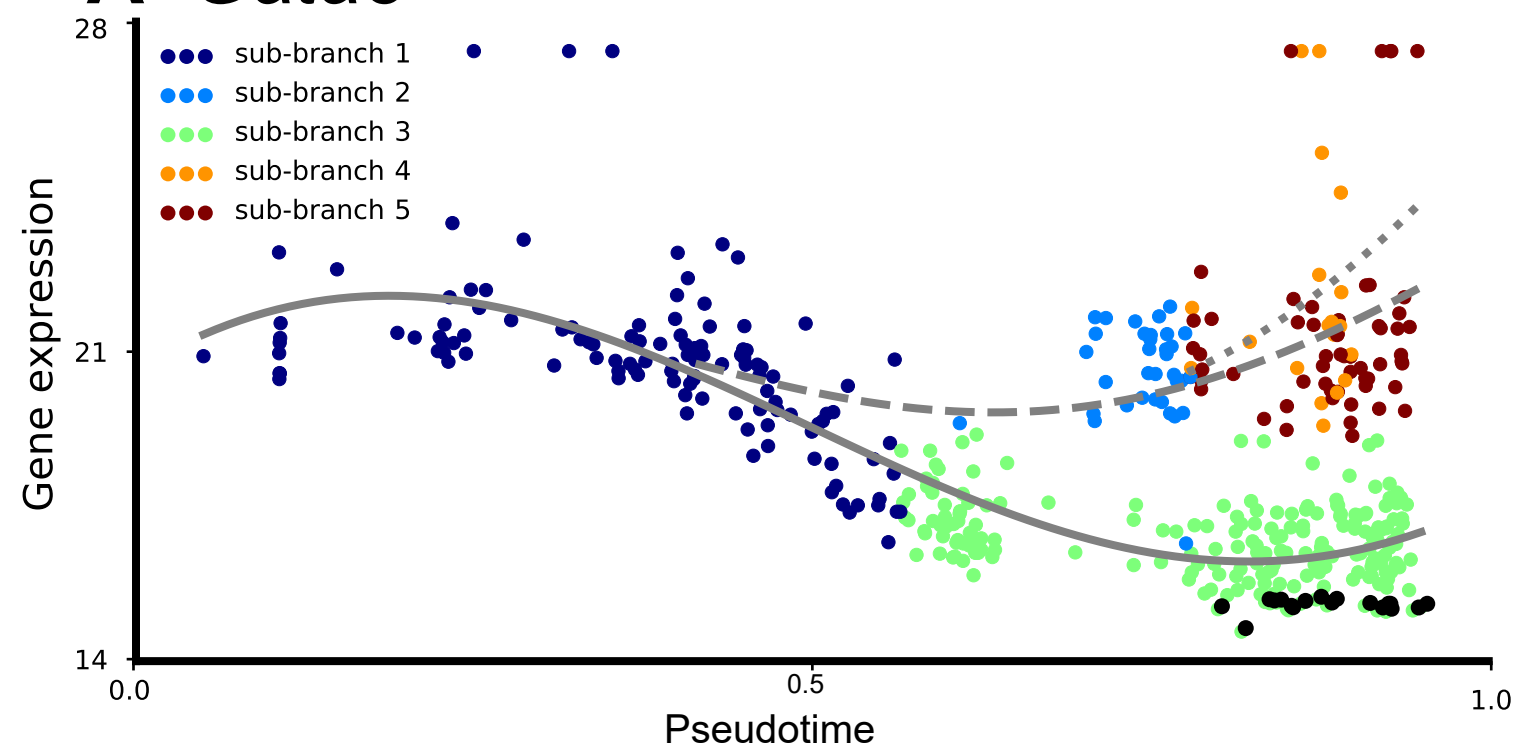**B** *Sox2*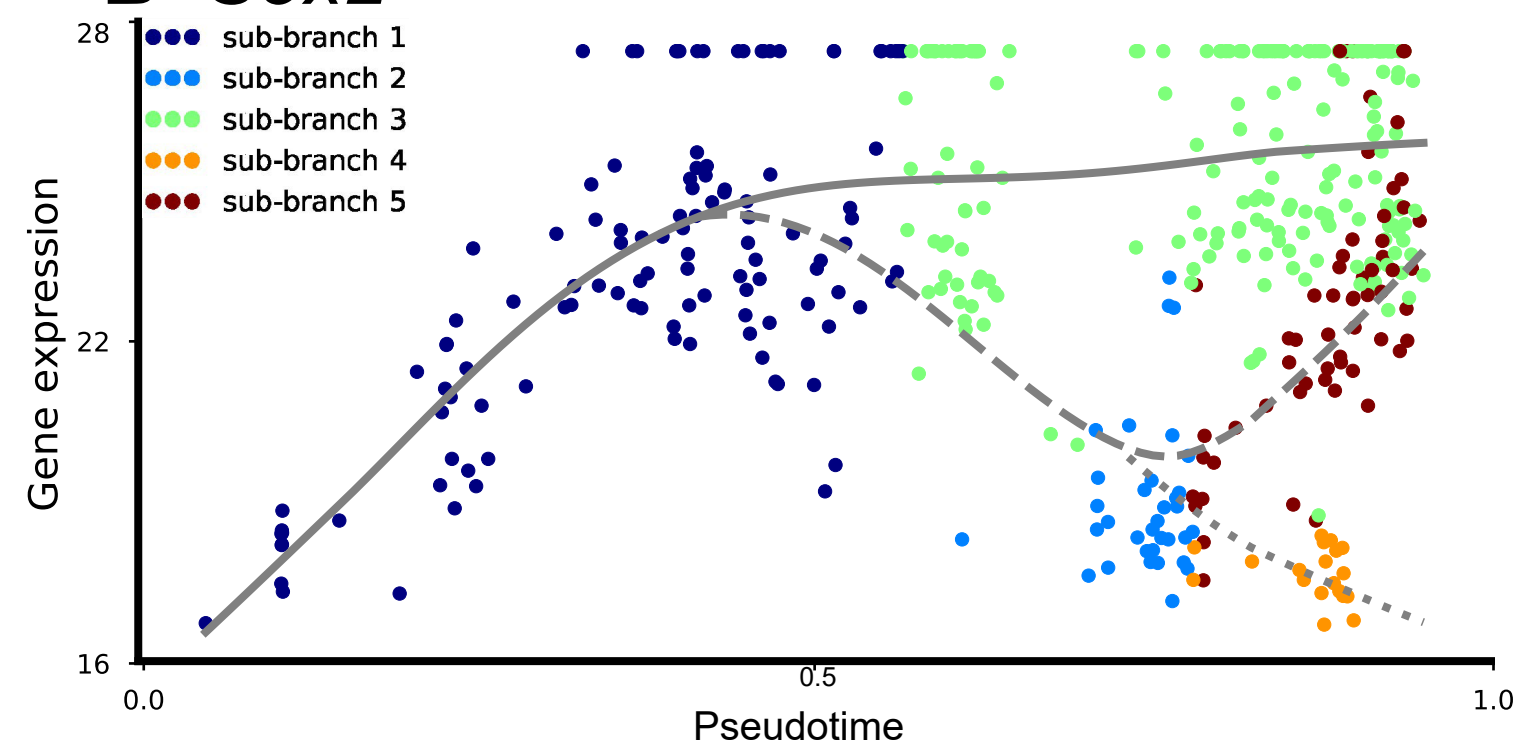

## Scanpy

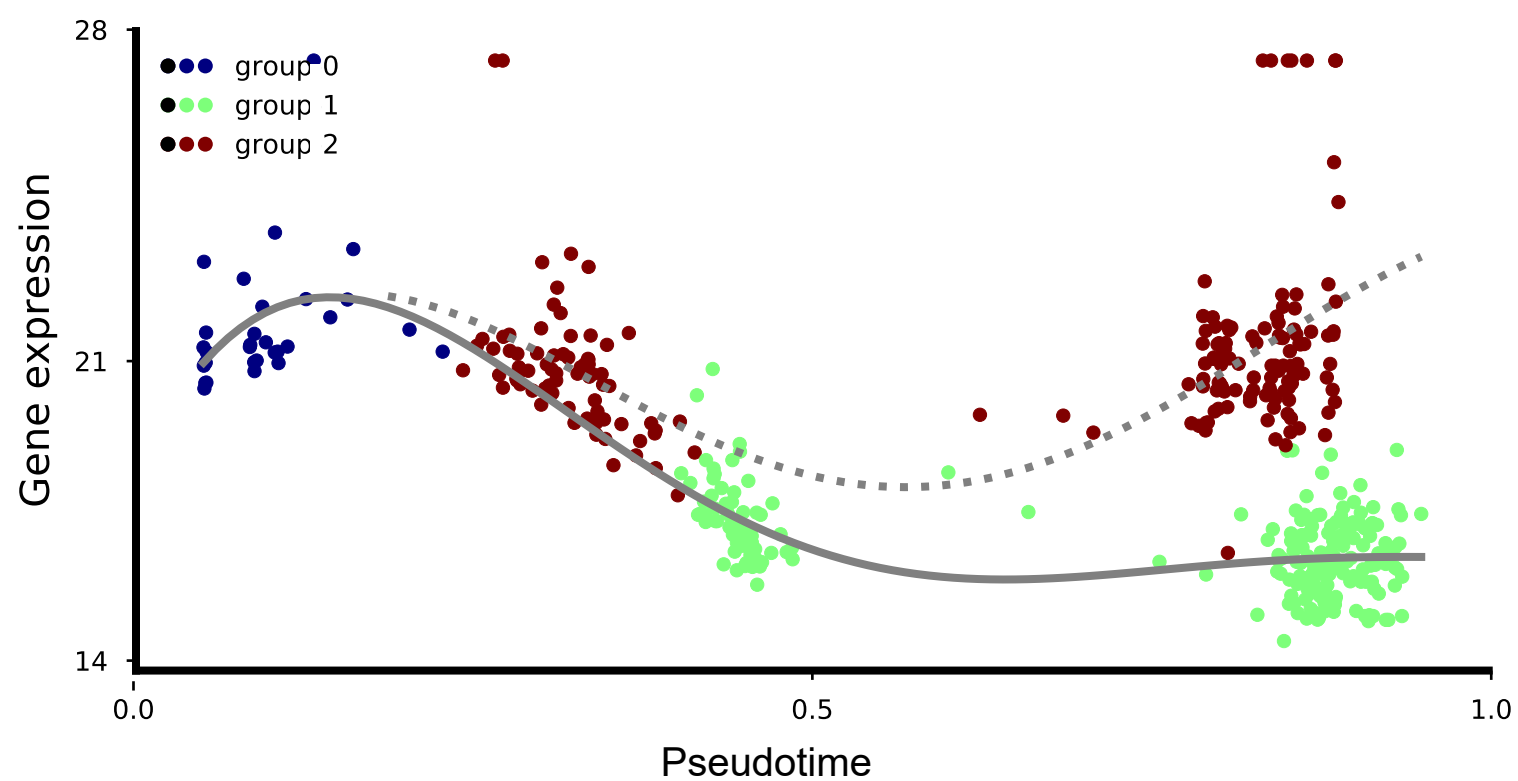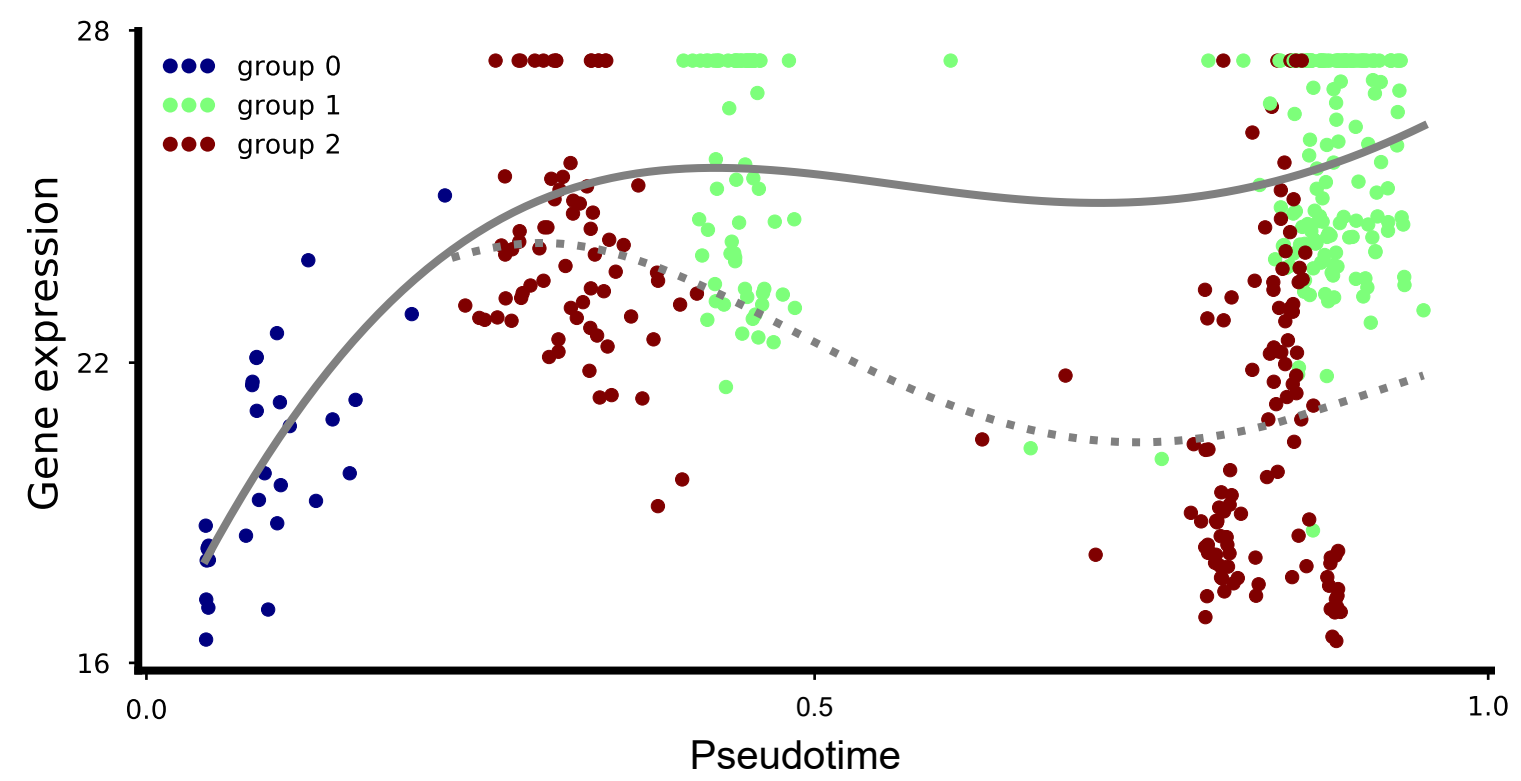

## Monocle

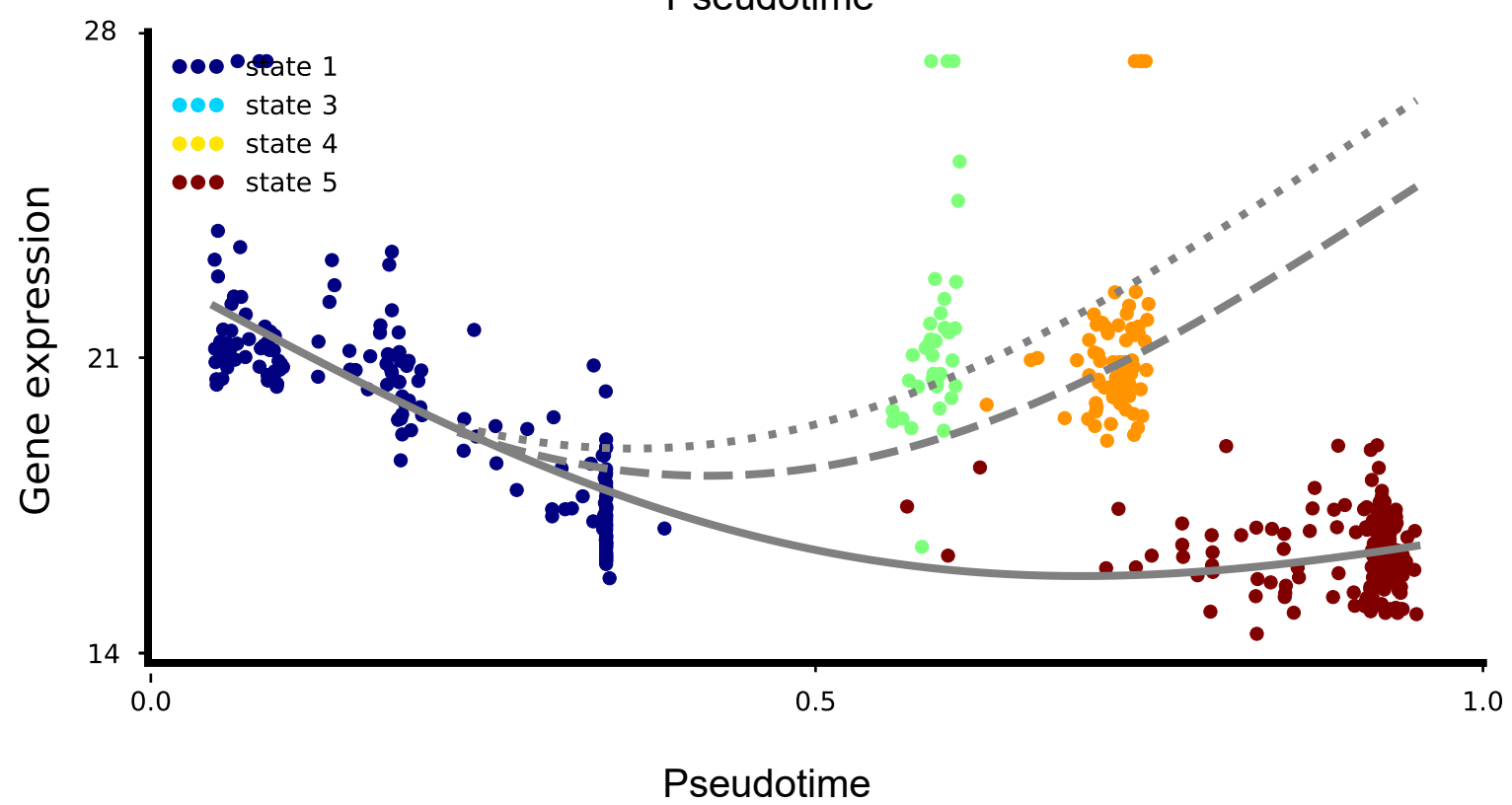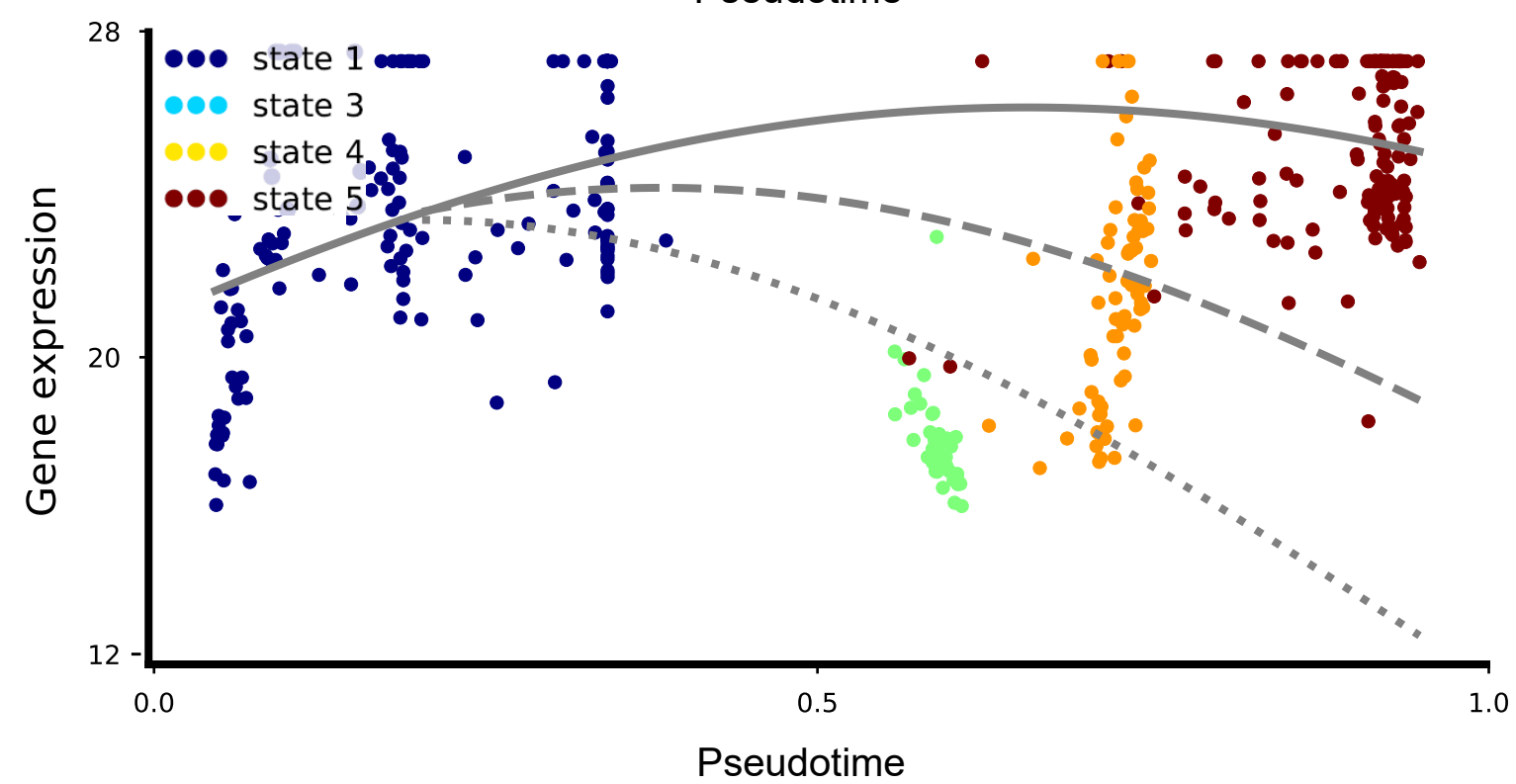

Supplement: Supplementary Figure S4 — Trajectories for the expression values of two genes of the ME dataset. A. Trajectories of gene Gata3 in the ME dataset with 48 genes and 438 single cells [43]. B. Trajectories of gene Sox2 in the ME dataset. The ordered values from the ME dataset are plotted along pseudotime obtained by using the three inference methods. The lines correspond to the results by using the Gaussion process regression for each branch. [file mmc5.pdf]

Robustness properties for ME dataset

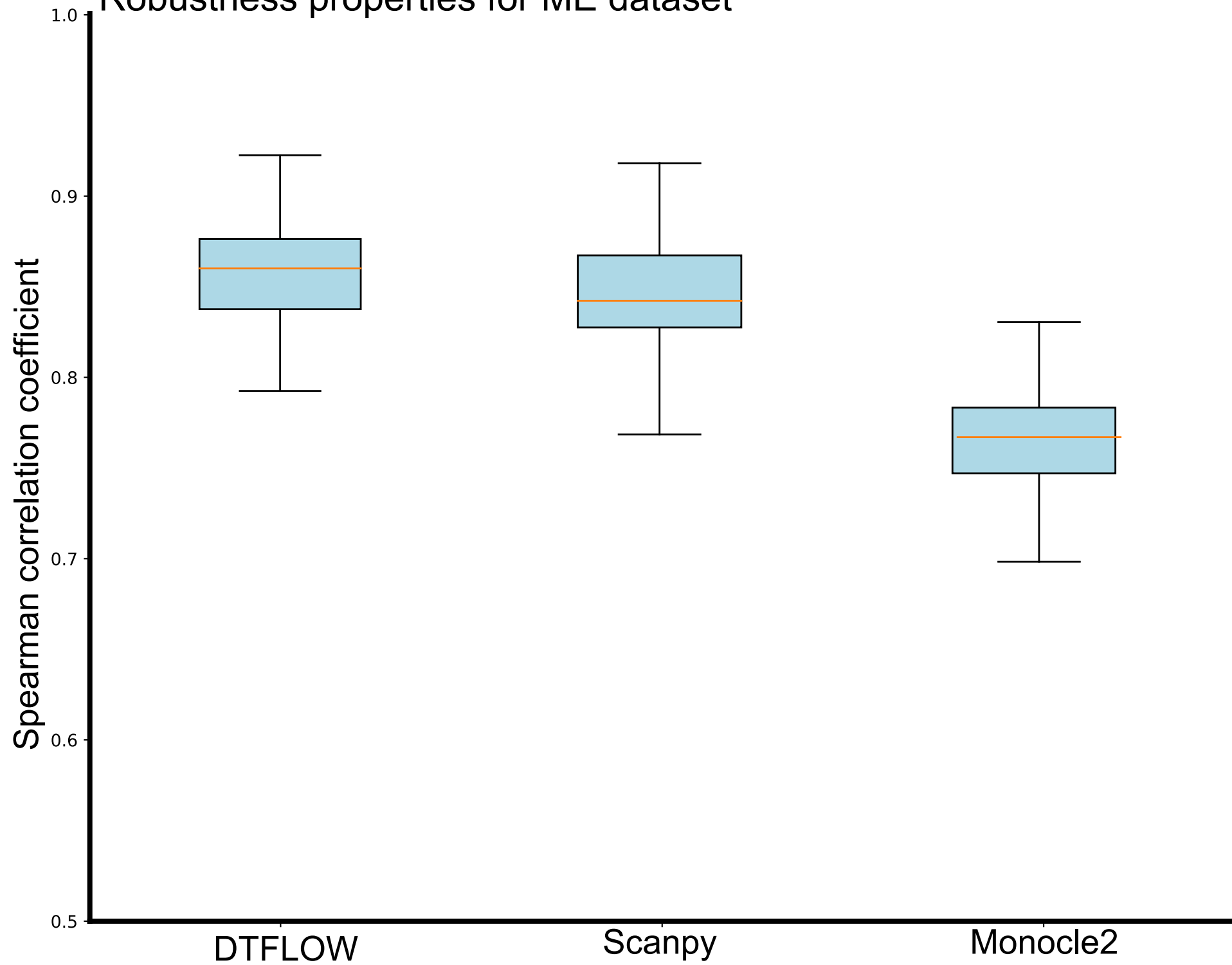

Supplement: Supplementary Figure S5 — Robustness properties of three inference methods for the ME dataset. We randomly sample 80% of single cells from the whole dataset with 48 genes and 438 single cells [43] and compare the ordering results of the subset with those of the whole dataset by using the Kendall rank correlation coefficient. Means and standard deviations of 50 repeated tests are presented. [file mmc6.pdf]

**A** Different time stages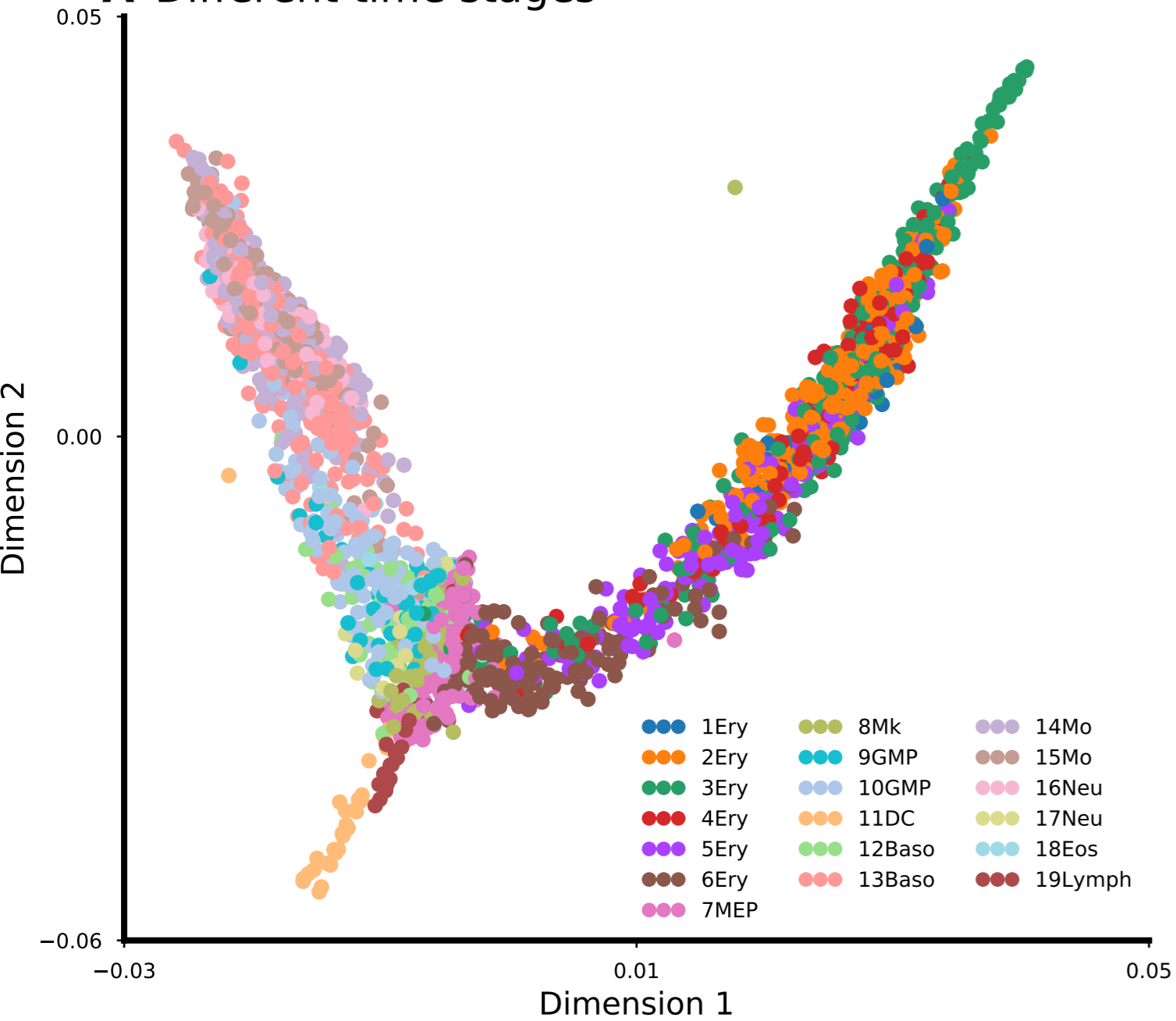**B** DPT pseudotime trajectory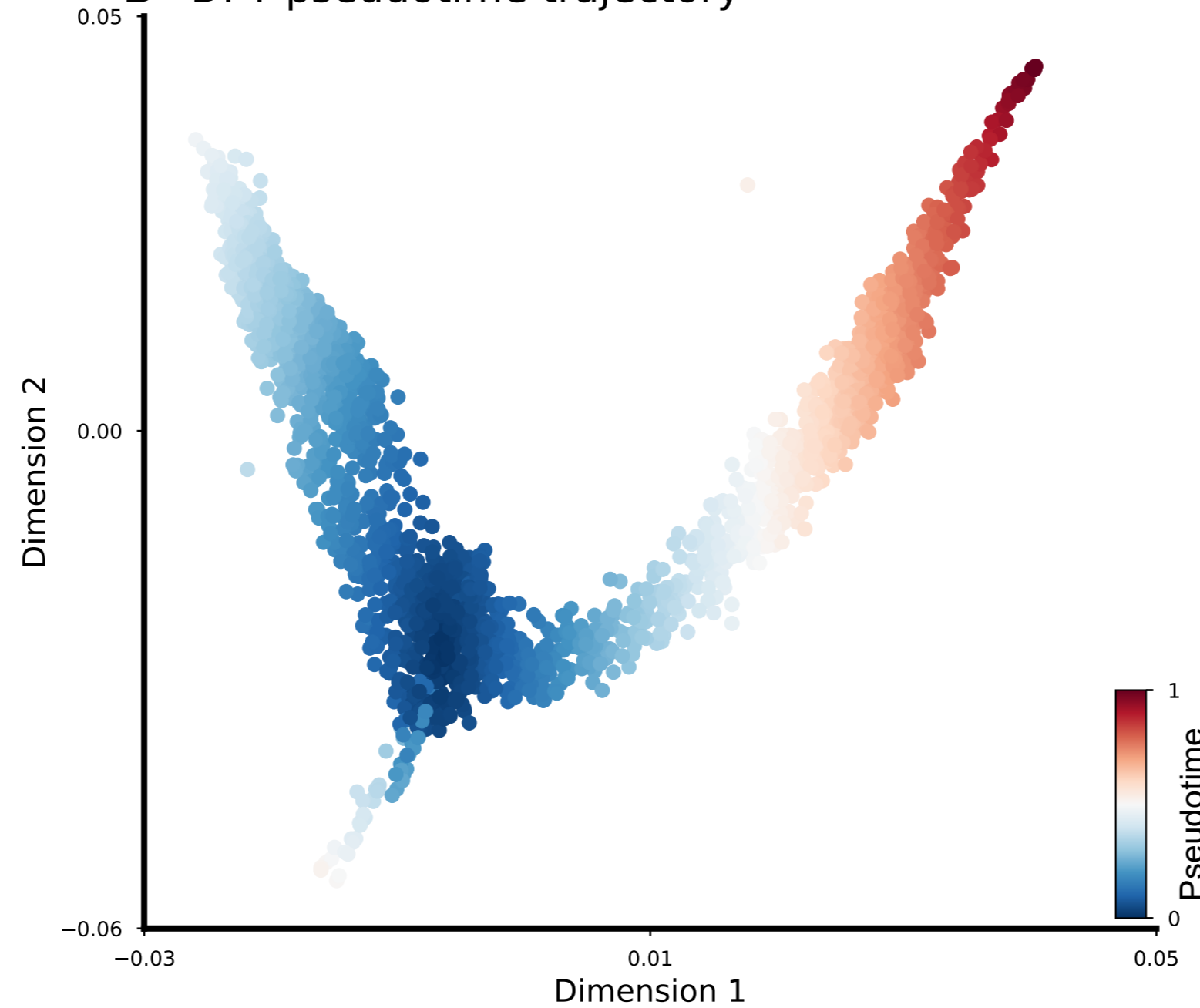**C** DPT groups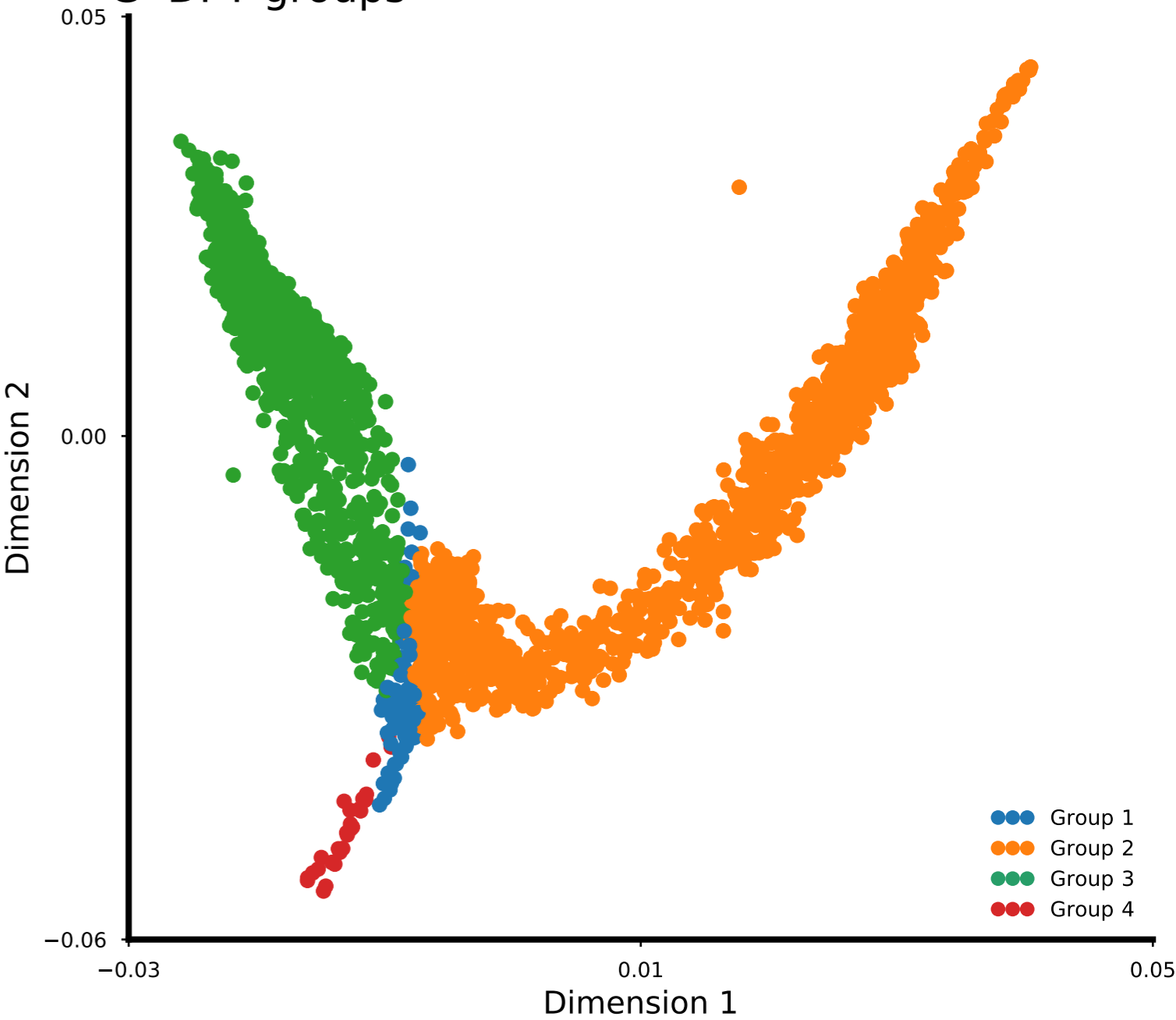

Supplement: Supplementary Figure S6 — Visualization of the MMP dataset using Scanpy. A. Visualization of 19 cell clusters in the MMP dataset with 3451 genes and 2730 single cells [44]. B. Visualization of pseudotime of each single cell, whose values range from 0 to 1. C. Visualization of 4 groups/sub-branches. [file mmc7.pdf]

**A** Different cell types

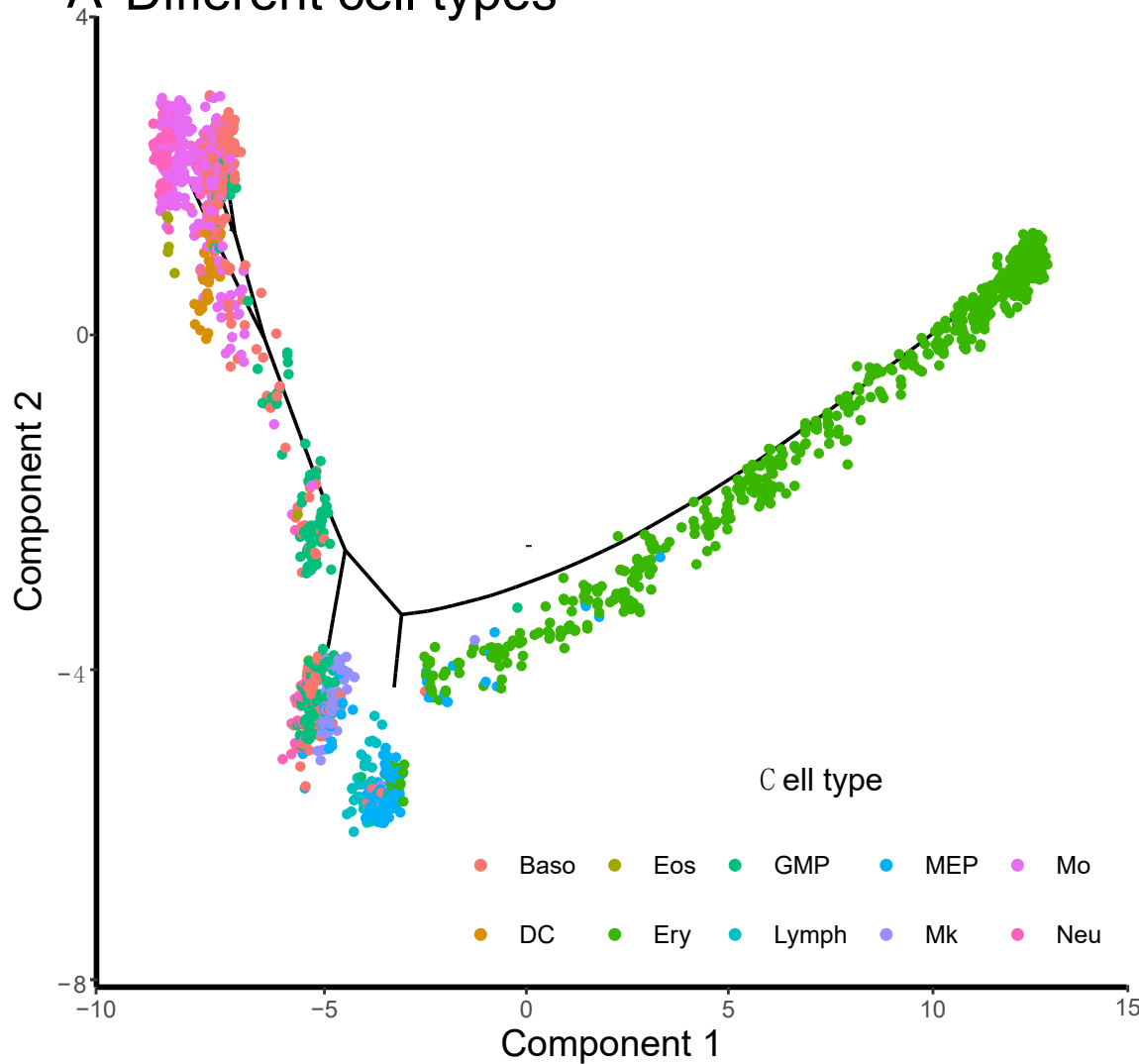

**B** Monocle pseudotime trajectory

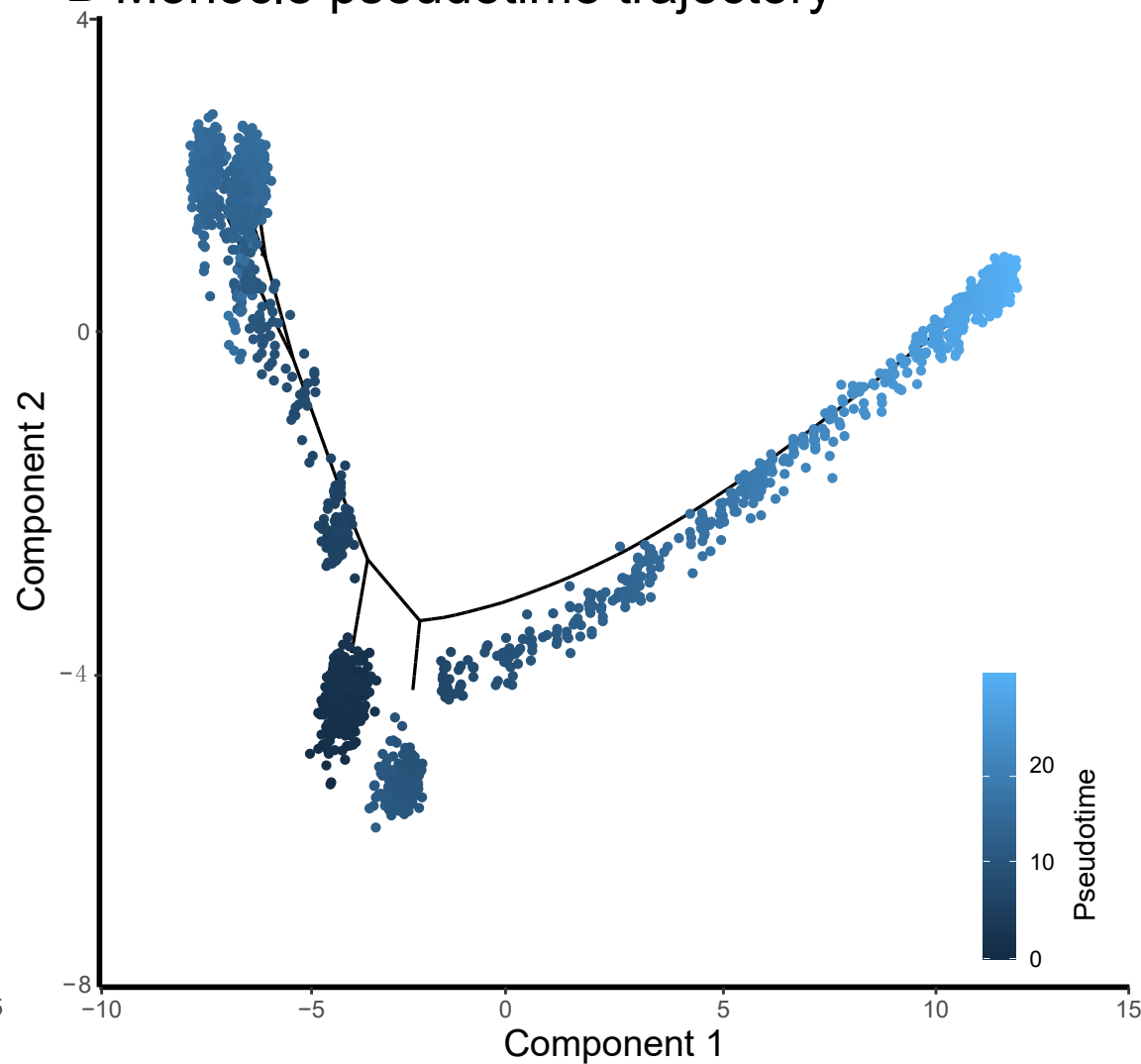

**C** Monocle states

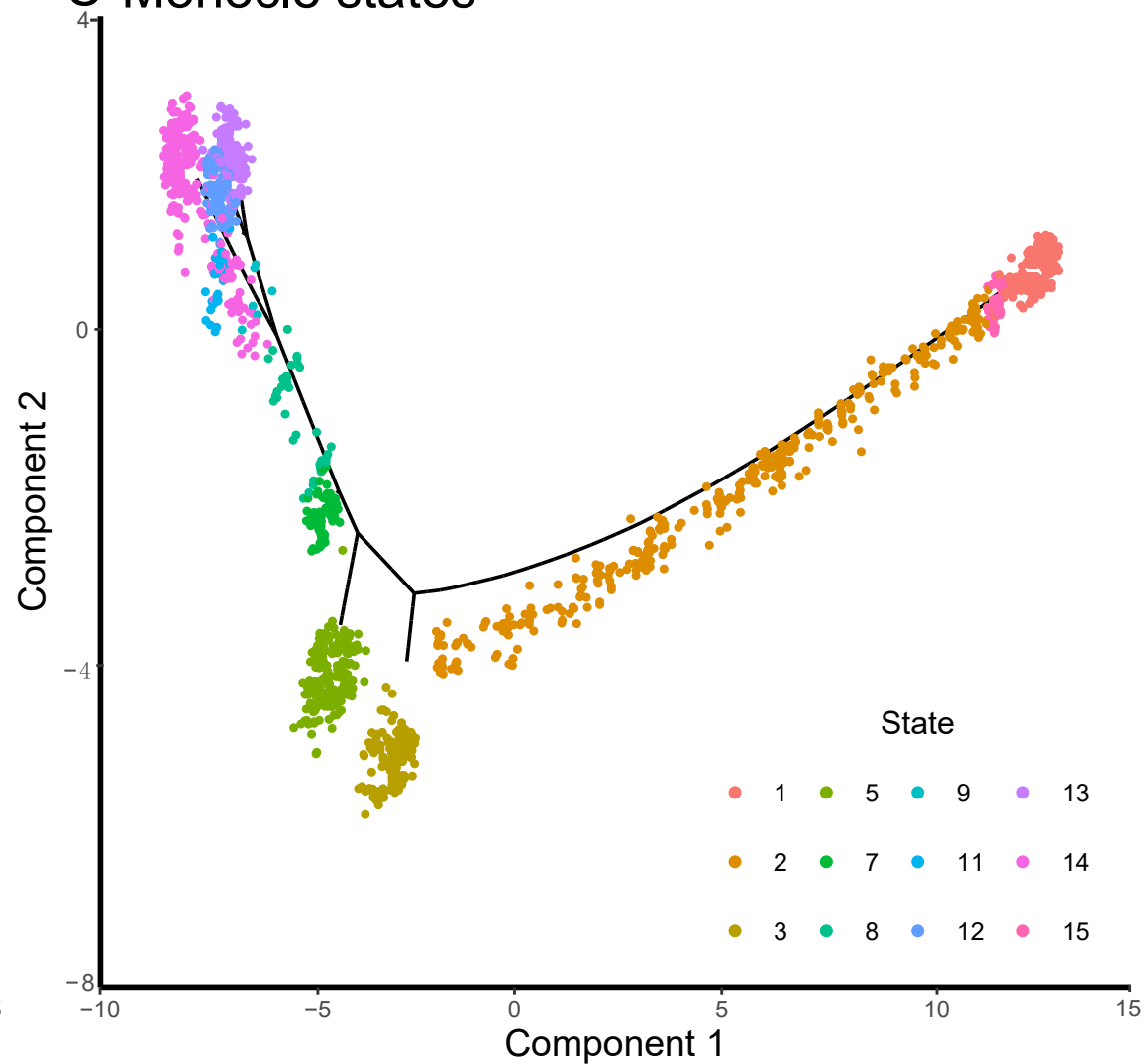

Supplement: Supplementary Figure S7 — Visualization of the MMP dataset using Monocle2. A. Visualization of 10 cell types in the MMP dataset with 3451 genes and 2730 single cells [44]. B. Visualization of pseudotime of each single cell, whose values range from 0 to 1. C. Visualization of 12 states/sub-branches. [file mmc8.pdf]

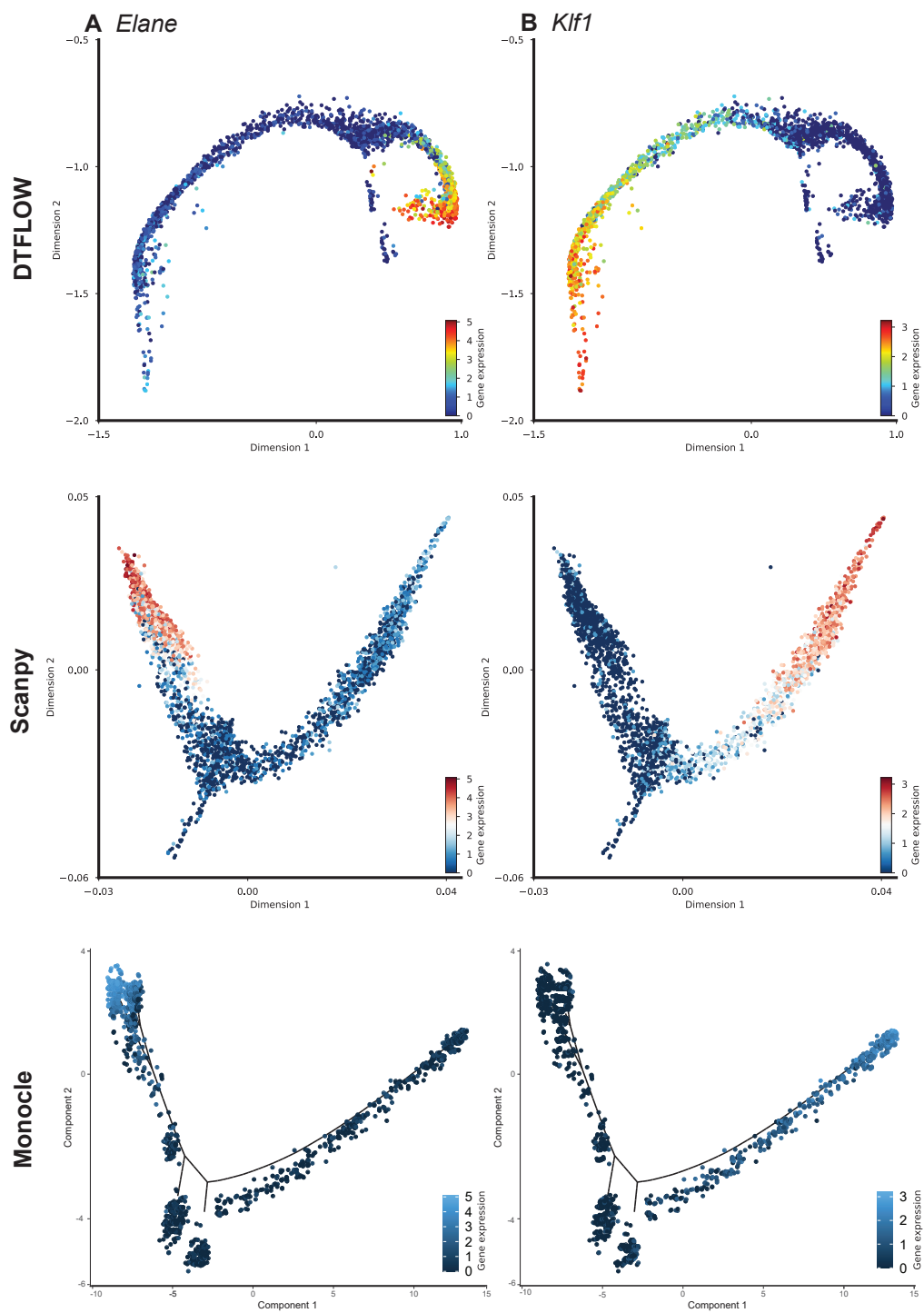

Supplement: Supplementary Figure S8 — Trajectories for the expression values of two genes of the MMP dataset. A. Trajectories of gene Elane in the MMP dataset with 3451 genes and 2730 single cells [44]. B. Trajectories of gene Klf1 in the MMP dataset. The ordered values from the MMP dataset are plotted along pseudotime obtained by using the three inference methods. The lines correspond to the results by using the Gaussian process regression for each branch. [file mmc9.pdf]

**A** Different time stages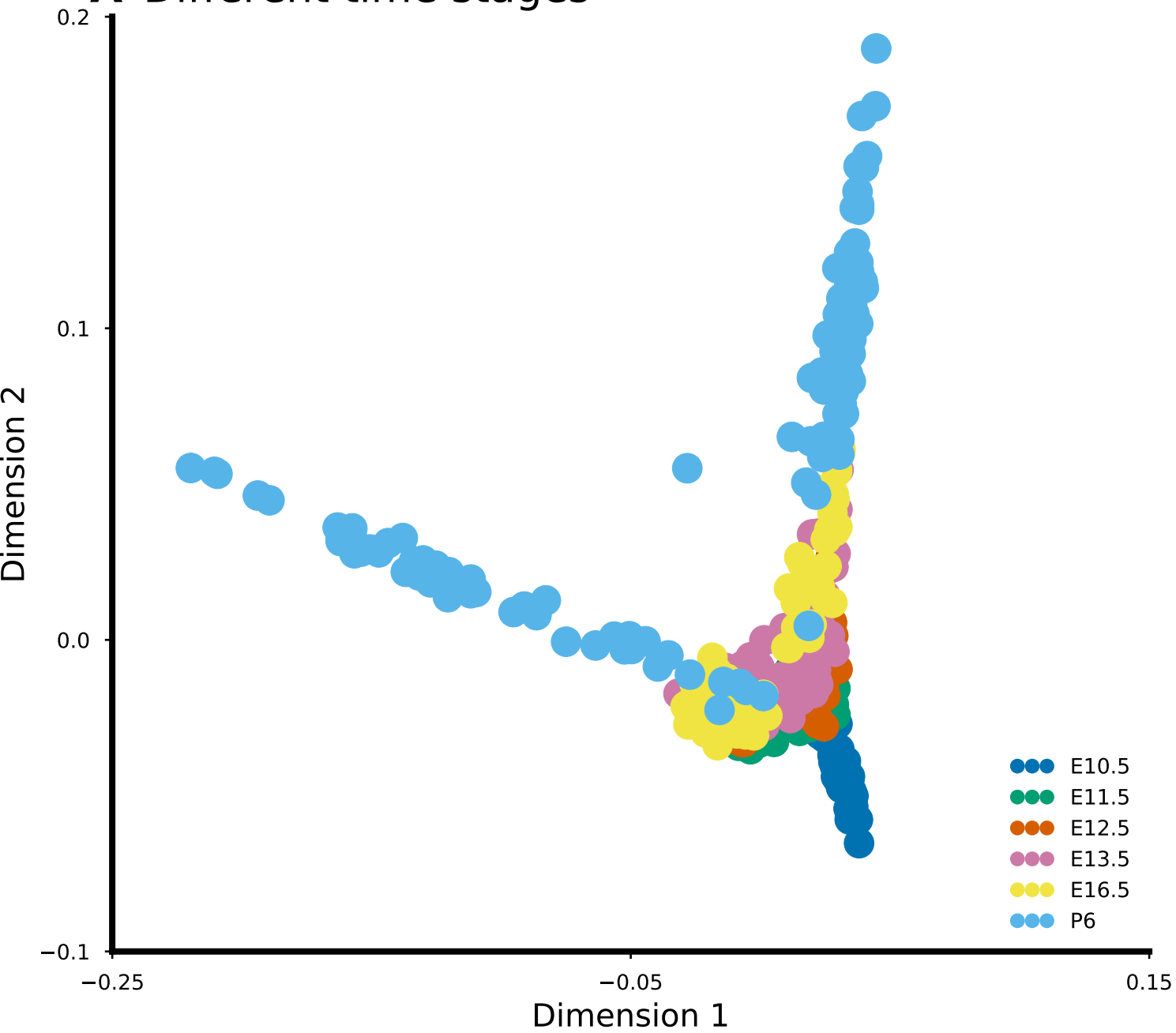**B** DPT pseudotime trajectory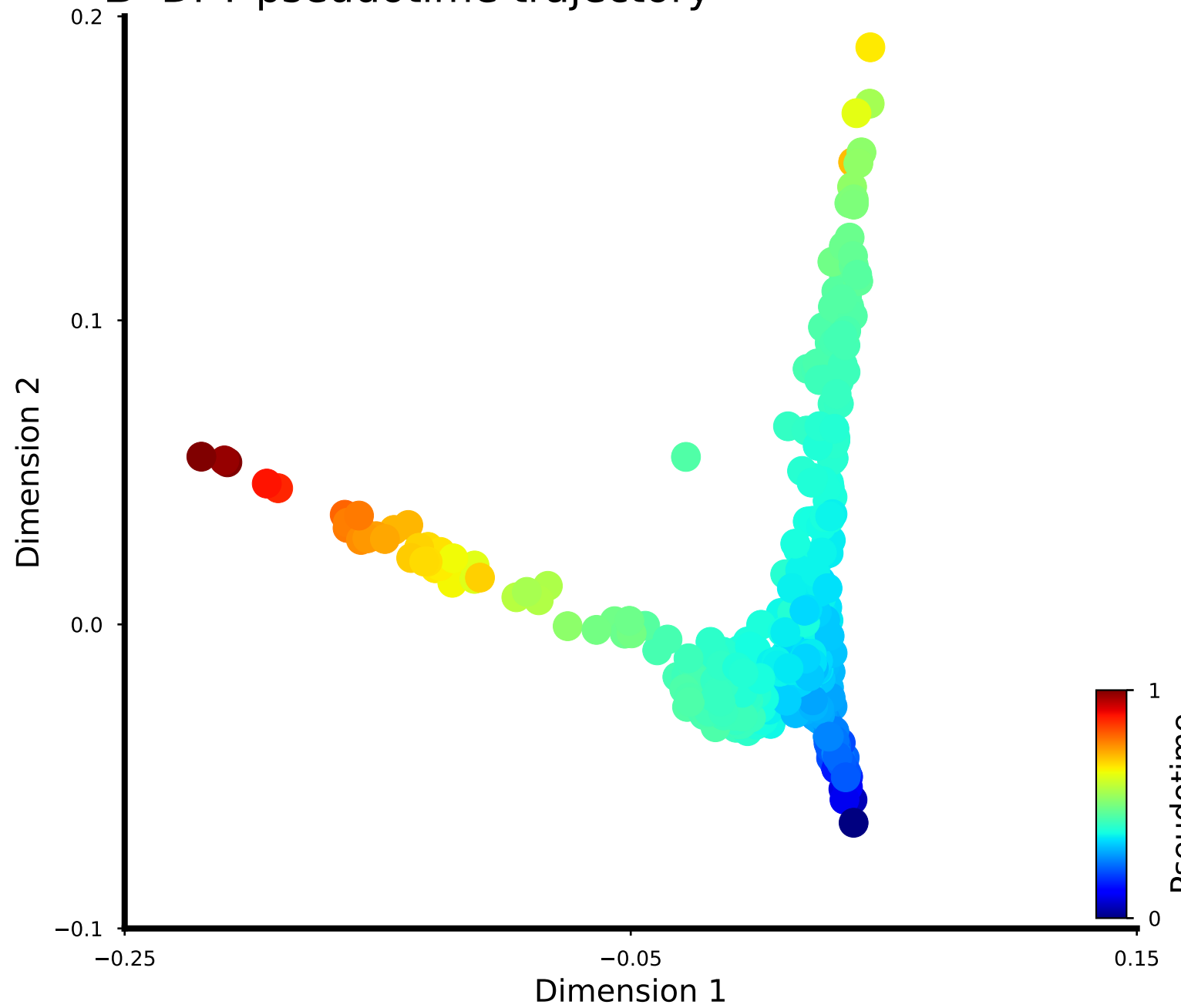**C** DPT groups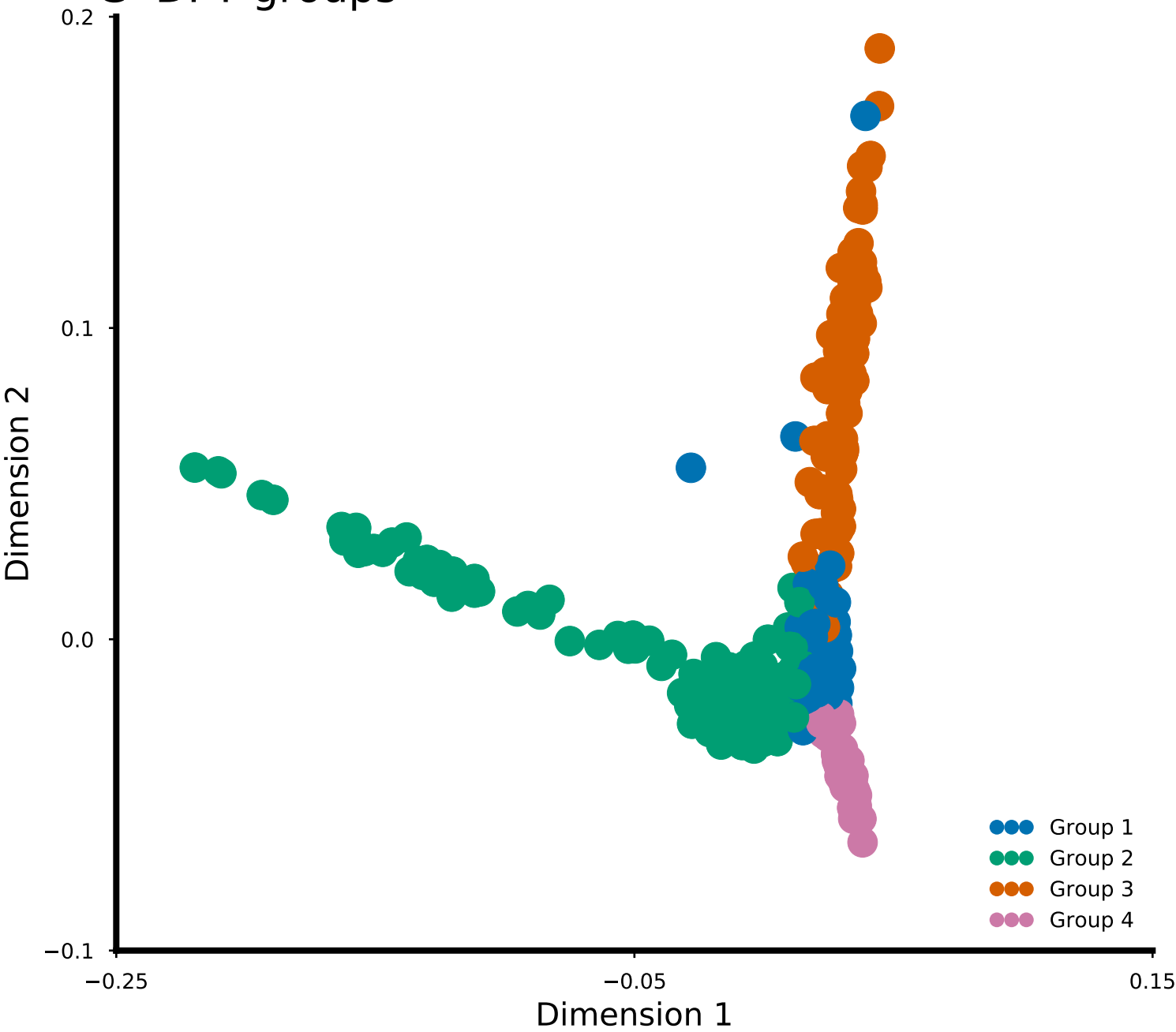

Supplement: Supplementary Figure S9 — Visualization of the MFG dataset using Scanpy. A. Visualization of 6 cell clusters in the MFG dataset with 822 genes and 563 single cells [45]. B. Visualization of pseudotime of each single cell, whose values range from 0 to 1. C. Visualization of 4 groups/sub-branches. [file mmc10.pdf]

**A** Different time stages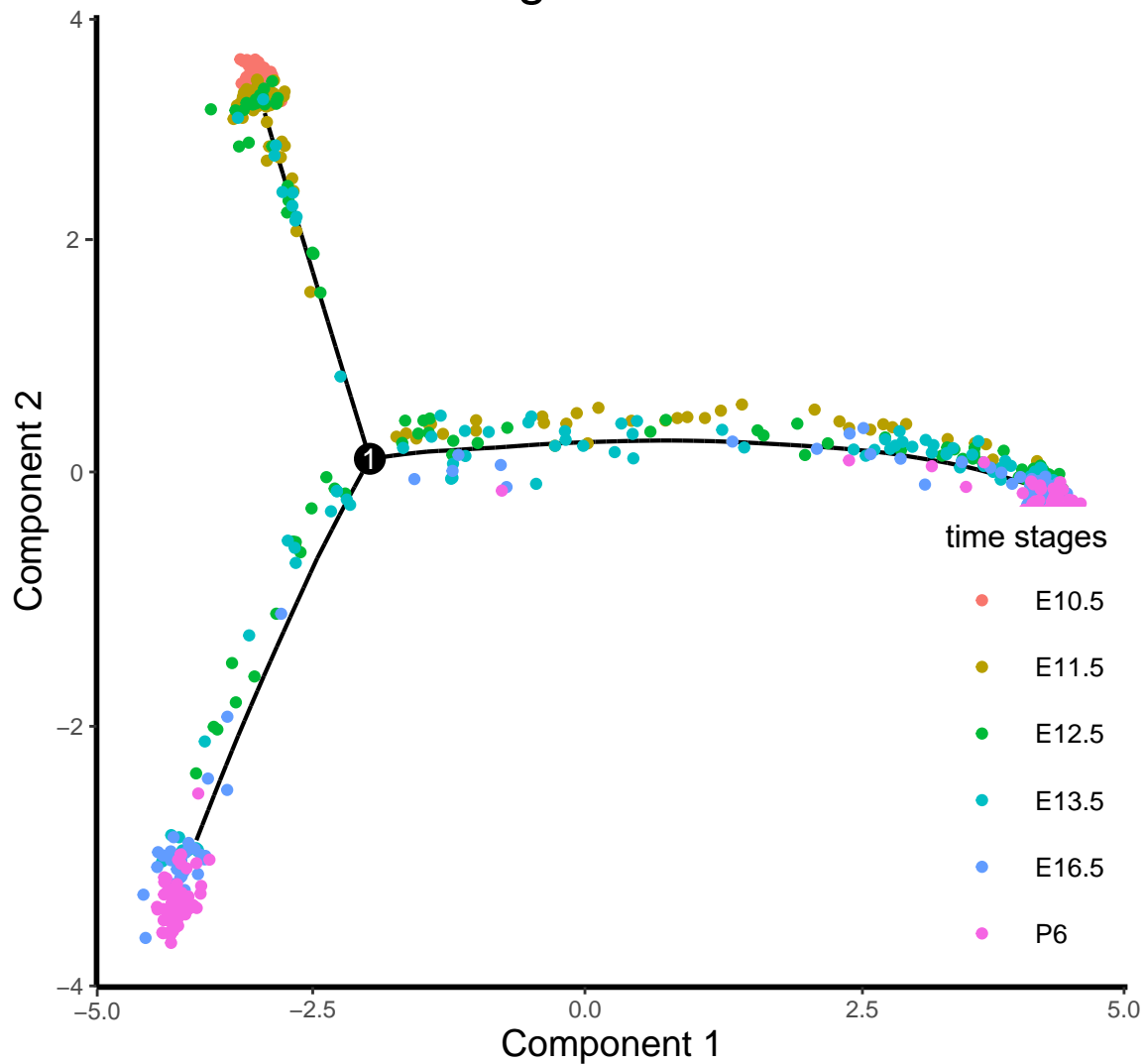**B** Monocle pseudotime trajectory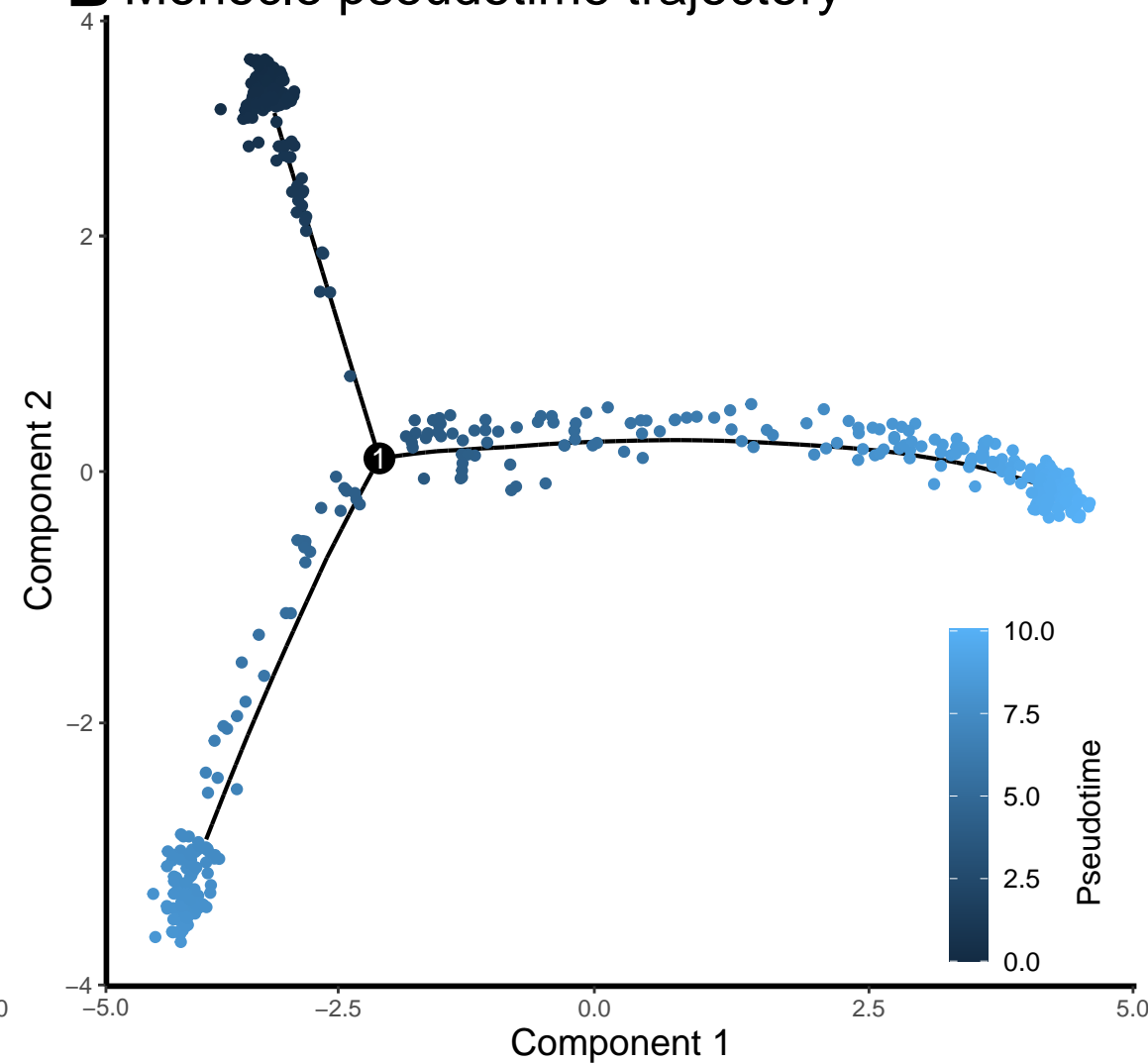**C** Monocle states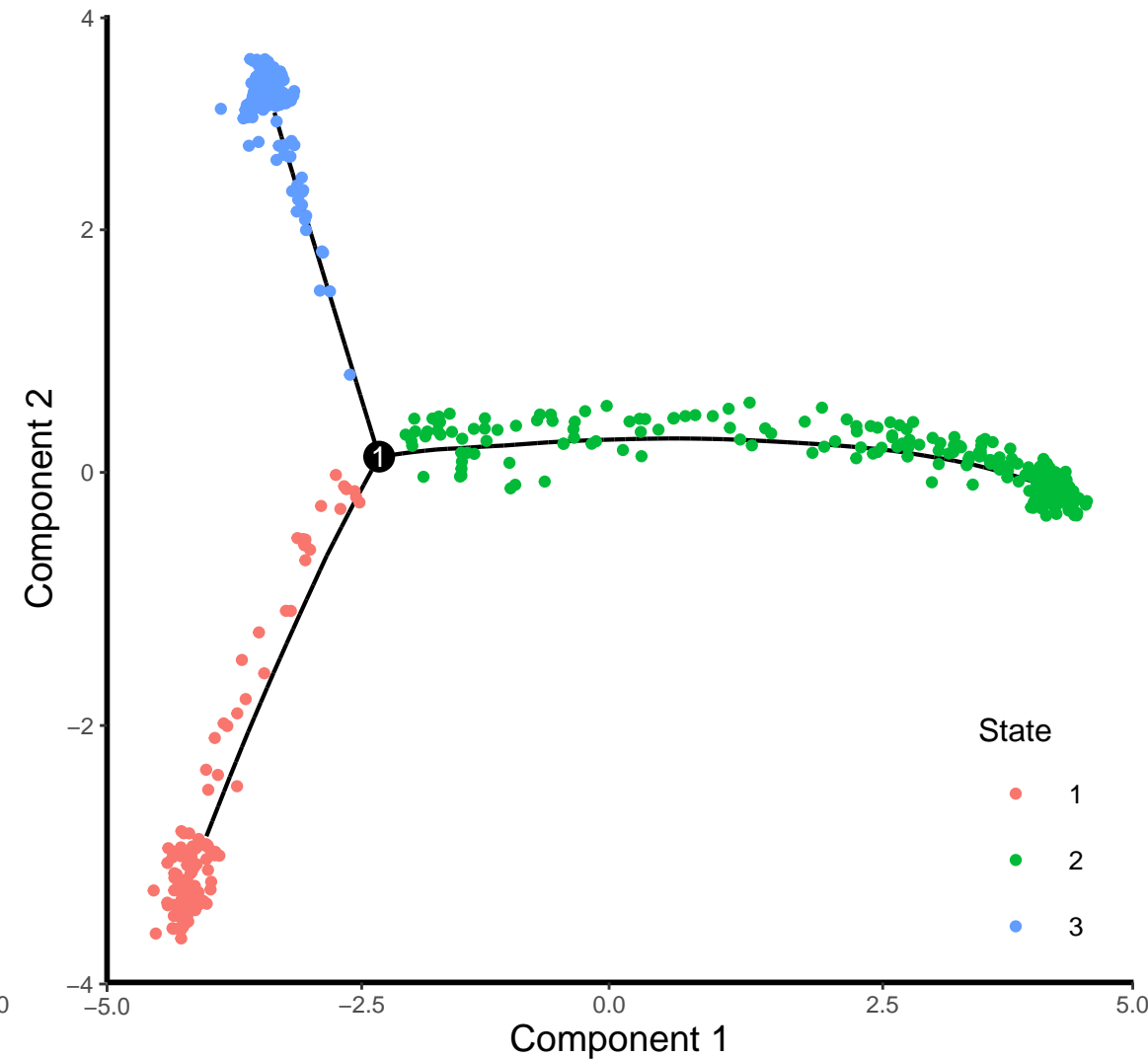

Supplement: Supplementary Figure S10 — Visualization of the MFG dataset using Monocle2. A. Visualization of 6 cell clusters in the MFG dataset with 822 genes and 563 single cells [45]. B. Visualization of pseudotime of each single cell, whose values range from 0 to 1. C. Visualization of 3 states/sub-branches. [file mmc11.pdf]
